# A role for metamemory in cognitive offloading

**DOI:** 10.1016/j.cognition.2019.104012

**Published:** 2019-12

**Authors:** Xiao Hu, Liang Luo, Stephen M. Fleming

**Affiliations:** a Collaborative Innovation Center of Assessment toward Basic Education Quality, Beijing Normal University, Beijing, China; b Wellcome Centre for Human Neuroimaging, University College London, London, United Kingdom; c Max Planck UCL Centre for Computational Psychiatry and Ageing Research, University College London, London, United Kingdom

**Keywords:** Cognitive offloading, Metacognition, Metamemory, Confidence, Memory, External memory

## Abstract

Cognitive offloading refers to our reliance on the external environment in order to reduce cognitive demand. For instance, people write notes on paper or smartphones in order not to forget shopping lists or upcoming appointments. A plausible hypothesis is that such offloading relies on metamemory – our confidence in our future memory performance. However, this hypothesis has not been directly tested, and it remains unclear when and how people use external sources to aid their encoding and retrieval of information. In four experiments, here we asked participants to learn word pairs and decide whether to offload some of the pairs by “saving” them on a computer. In the memory test, they had the opportunity to use this saved information on half of trials. Participants adaptively saved the most difficult items and used this offloaded information to boost their memory performance. Crucially, participants' confidence judgments about their memory predicted their decisions to use the saved information, indicating that cognitive offloading is associated with metacognitive evaluation about memory performance. These findings were accommodated by a Bayesian computational model in which beliefs about the performance boost gained from using offloaded information are negatively coupled to an evaluation of memory ability. Together our findings highlight a close link between metamemory and cognitive offloading.

## 1 Introduction

Learning often relies on external tools and resources. For example, when we attend lectures in school, we write things down into a notebook rather than relying on memory alone because we believe a written record is more reliable. Following a rapid development in modern technology, we now also save information into our smartphones or computers to reduce memory load. However, we still lack an understanding of when and how people rely on external sources to facilitate memory. In the current paper we pursue a hypothesis that use of external resources is intimately linked to metacognitive evaluations of memory performance (Arango-Muñoz, 2013, Finley et al., 2018, Risko and Gilbert, 2016).

Human learners are able to self-regulate behavior to optimize memory performance (for a review, see Bjork, Dunlosky, & Kornell, 2013). For instance, previous studies indicate that people tend to restudy previously unrecalled rather than recalled items and spend more time learning difficult than easy items (Metcalfe and Finn, 2008, Metcalfe and Kornell, 2005). These strategies can efficiently improve memory (Bjork et al., 2013). However, fewer studies have investigated a distinct, but potentially equally important type of self-regulation – our reliance on external storage (Risko & Dunn, 2015). For example, we might write down crucial information such as a phone number or type it into a smartphone, and then later attempt to find the relevant information (Risko and Dunn, 2015, Storm and Stone, 2015). This strategy is known as “cognitive offloading”, which refers to the use of physical actions to alter the information processing requirements of a task (Risko & Gilbert, 2016).

The aim of the present study is to shed light on when and how people use external tools to aid their learning, by developing and empirically validating a computational model that precisely characterizes individuals’ use of offloaded information. Compared with descriptive theories, computational models are able to quantitatively characterise a hypothesised cognitive process and provide insights that are otherwise difficult to obtain from traditional behavioural data analysis (Lewandowsky & Farrell, 2011). In doing so we focus on the potential link between metacognition and offloading, as it is plausible that our decisions of *when* to rely on outside assistance depend on metacognitive appraisal of expected performance (Arango-Muñoz, 2013, Finley et al., 2018, Risko and Gilbert, 2016).

Cognitive offloading reduces cognitive demand on memory because it reduces demands on internal storage. Rather than encoding individual items, people only need to remember the location of the saved items (Risko and Gilbert, 2016, Wegner, 1987). Previous studies have shown that allowing people to offload information can significantly improve performance on short-term and prospective memory tasks (Gilbert, 2015a, Gilbert, 2015b, Risko and Dunn, 2015). However, it remains unknown *how* people choose to offload cognitive demands during encoding and retrieval (Risko & Dunn, 2015). In the current study, we asked participants to learn a series of word pairs and decide whether to “save” the pairs to the computer. In a later memory test, on half of the trials participants had the opportunity to use the offloaded information, whereas on the other half of trials it was unavailable. This design allowed us to examine whether cognitive offloading improves long-term memory performance and, critically, establish how participants choose to use offloaded information during study and test.

According to a metacognitive model, whether people choose to offload to-be-remembered items depends on metacognitive evaluation (Dunn and Risko, 2016, Risko and Gilbert, 2016). Drawing on metacognitive beliefs and experiences about internal and external memory storage, people choose the option that they believe will lead to higher memory performance. The metacognitive model suggests that whether people choose to offload to-be-remembered information should depend on memory load. Higher memory load results in greater perceived difficulty of remembering, leading participants to opt for external storage to reduce internal demands. Consistent with this hypothesis, Risko and Dunn (2015) found that in a short-term memory task, as the number of to-be-remembered items increased, the likelihood of offloading behavior increased. Similarly, Gilbert (2015a) found that in a prospective memory task, when participants had to keep in mind specific task requirements for three items compared to one item, they tended to set more frequent external reminders. Besides the number of items, the decision to offload may also be related to item difficulty: people should offload individual items more frequently when they are more difficult to remember (Schönpflug, 1986). In the present study, we manipulated item difficulty to examine this influence on offloading behavior.

After deciding to offload information to the environment, people must then decide whether to access the offloaded information. In the current study we also sought to model the factors that influenced these decisions. One possibility is that people decide to rely on offloaded information when they believe that they cannot retrieve the items by themselves, or when they have low confidence about retrieving particular items. Previous studies on help-seeking behaviour reveal that humans and animals tend to seek help from external sources when their confidence on a task is low, and rely more on their own abilities when their confidence is high (Desender et al., 2018, Goupil et al., 2016, Kornell et al., 2007). Thus, similar to the offloading decisions made during encoding, whether people decide to use offloaded information should also depend on metacognitive evaluation of self-performance. In addition, when people decide whether to rely on offloaded information, they should evaluate not only their memory for individual items, but also the extent to which they believe offloaded information will improve memory performance. In the current study, we examined the determinants of participants’ decisions to use offloaded information and (in Experiments 2 and 3) how such decisions related to their metacognitive evaluation (confidence) about how much offloaded items would boost their performance. We sought to accommodate these findings within a novel probabilistic computational model that precisely characterizes how people decide to use offloaded information during memory retrieval.

To anticipate our results, across four experiments, we show that allowing participants to use offloaded information significantly enhanced their memory performance. The difficulty of individual items affected participants’ decisions to use offloading strategies at both encoding and retrieval. Our computational model indicated that participants’ beliefs about the boost in performance they would gain from offloaded information was negatively correlated with evaluations of their memory ability, which in turn guided their decisions to use offloaded information during retrieval. Our findings support a close link between cognitive offloading and metacognition about memory performance.

## 2 Experiment 1

### 2.1 Methods

#### 2.1.1 Participants

Twenty-seven participants (8 men; age: M = 24.85 years, SD = 9.19) took part in the experiment at the Wellcome Centre for Human Neuroimaging, University College London for monetary compensation (£6 plus up to £2 bonus, dependent on performance in the memory test). Participants were tested individually, and all provided written informed consent. All participants spoke English as a first language and reported normal or corrected-to-normal vision. All procedures were approved by the local ethics committee.

#### 2.1.2 Materials

The study materials consisted of 120 cue-target English word pairs. All of the words were 4–8 letters in length and from the MRC Psycholinguistic Database (Coltheart, 1981). Of these word pairs, half were easy pairs in which cue and target words were strongly related, and half were difficult pairs in which cue and target words were unrelated. The easy and difficult pairs significantly differed in the forward associative strength from cue to target words, which represents the proportion of participants in the original norming study who first responded with the target word when free associating to the cue word (Kiss, Armstrong, Milroy, & Piper, 1973). The forward associative strength was significantly higher for easy pairs (M = 0.43, SD = 0.13) than for difficult pairs (0 for all of the difficult pairs) according to the Edinburgh Associative Thesaurus (Kiss et al., 1973). The number of letters, number of syllables, word frequency and ratings of familiarity, concreteness and imagability for cue and target words did not differ between easy and difficult pairs, all *t*s < 1, all *p*s > 0.3. All of the word pairs were randomly divided into three lists, and each list contained 20 easy and 20 difficult pairs. Another 12 word pairs (6 easy and 6 difficult pairs) were used in the practice block. Please see Table S1 in the Supplemental Information for the word pairs used in the current study.

#### 2.1.3 Procedure

**Fig. 1:**
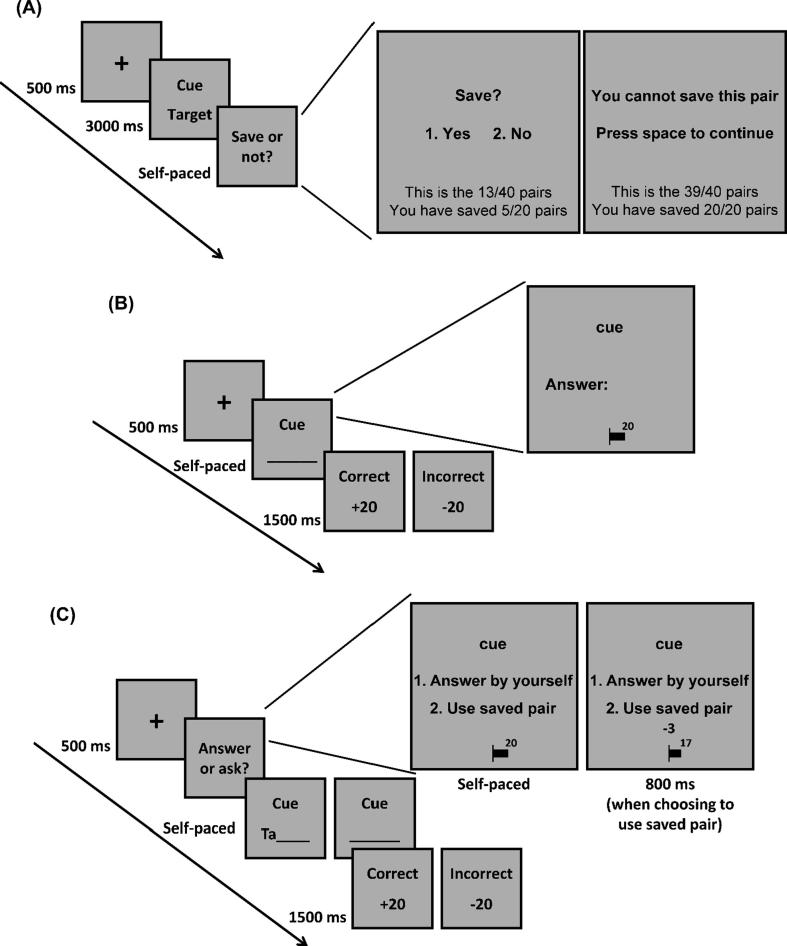
Experimental procedure for the task in Experiment 1. (A) Learning phase. (B) Forced-recall test. (C) Free-choice test.

Stimulus presentation and data acquisition were controlled by Psychtoolbox (Brainard, 1997) running in MATLAB (http://www.mathworks.com). The experiment contained three blocks. In each block, participants learned a list of 40 word pairs and then took a memory test for that list. The order of the lists was randomised across participants. Before the experiment, participants read the instructions and completed a practice block in which they were familiarized with the full experimental procedure with 12 example word pairs. The procedure of the learning phase is shown in Fig. 1A. During the learning phase of each block, participants were required to study 40 word pairs (20 easy and 20 difficult pairs) sequentially. The word pairs were presented in a pseudo random order in which no more than three word pairs of the same difficulty were presented consecutively. In each trial, a fixation cross was first presented for 500 ms, and then a word pair was presented in the center of the computer screen for 3 s. Immediately following the presentation of each word pair, participants were asked to decide whether to save this pair into the computer by pressing the 1 or 2 key. Before the experiment, participants were informed that they could use the saved pairs to help them recall the words in the memory test. In each block, they could save at most half of the list (i.e., 20 pairs) into the computer. When they decided whether to save each pair, they could see how many pairs had been presented and how many had been saved so far. If participants had saved half of the list before the end of the list, then they could not save the remaining pairs in the list and could only press the space bar to continue after the presentation of the word pair. In data analysis, we removed trials in which participants could not save the word pair (around 1% of the trials).

Participants took a memory test immediately after they finished learning each list. There were two types of memory test: a forced-recall test and a free-choice test. For each participant, the saved and unsaved trials during learning were randomly assigned to the two test conditions so that participants took the forced-recall test for half of the saved and unsaved trials, and the free-choice test for the other half. The trials in the two test conditions were randomly intermixed in the test phase with the constraint that no more than three trials of the same type of test were presented consecutively.

The procedure of the forced-recall and free-choice test is shown in Fig. 1B and 1C. In the forced-recall test. a fixation was first presented for 500 ms, and then the cue word of a pair was presented on the screen. Participants were instructed to use the keyboard to type the target word and then press the Enter key to submit their answer. There was no time limit for the recall test. After participants submitted the answer, feedback was presented for 1.5 s, showing the correctness of the answer and the points that were won or lost. For each trial, participants gained 20 points if their answer was correct, or lost 20 points if the answer was incorrect. In the memory test, a bar showing the total points earned was always presented on the screen. The points were converted into a monetary bonus awarded after the experiment.

In the free-choice test, the cue word of each pair was presented after fixation, and participants were asked to decide whether to recall the target word by themselves or obtain a hint generated from the saved pairs (“ask for help” trials). If they chose to answer by themselves, they were prompted to type the target word and feedback showed the correctness of their answer and the points won or lost. A choice to ask for help from the computer led to an immediate loss of 3 points, which was shown on the screen for 800 ms. If the word pair had been saved during learning, the first two letters of the target word were presented on the screen as a hint, and participants only needed to type the remaining letters of the target word. However, if the pair had not been saved, participants were prompted to type the whole target word without a hint. Participants lost 3 points for the choice to ask for help regardless of whether the word pair had been saved. This loss of 3 points was introduced to dissuade them from asking for help on every trial, incentivising asking for help only when the benefit of doing so (in terms of improving memory performance) outweighed this cost. After participants submitted their answers, feedback was presented showing the correctness of their answer and points earned. Participants were shown the total points they earned at the end of the experiment. In addition, both before and after the experiment, they were shown how these points were converted into a monetary bonus (see Table S2 in the Supplemental Information).

#### 2.1.4 Design

Experiment 1 used a within-participant design. In the learning phase, the dependent variable was the proportion of saved word pairs during learning, and the independent variable was item difficulty (easy vs. difficult). In the test phase, there were two dependent variables: recall performance and the proportion of trials in which participants asked for help in the free-choice test. For this ask-for-help proportion, the independent variables were item difficulty and whether the word pair was saved during learning (saved vs. unsaved). For recall performance, the independent variables were item difficulty, saved/unsaved pair and test type (forced-recall vs. free-choice). Participants could rely only on their memory to recall the target words in the forced-recall test, while in the free-choice test they could decide whether to use the offloaded information from the computer. We compared recall performance in the forced-recall and free-choice tests to examine whether allowing participants to use offloaded information would significantly improve their memory.

#### 2.1.5 Data analysis

The data from three blocks were collapsed in the analysis. We used t-tests to analyse the effect of item difficulty on the decisions to save the word pairs during learning. To analyse the effects of item difficulty, whether the word pair was saved and test type (forced-recall/free-choice) on the decisions to ask for help and recall performance in the memory test, we built linear mixed effect models in SPSS 22 to accommodate missing data in some conditions for some participants. For example, some participants did not save any easy word pair during learning. Traditional repeated measures analysis of variance (ANOVA) uses listwise exclusion, and none of the data from a participant can be used if data from one condition is missing. In contrast, linear mixed effect models can handle missing data and incorporate all of the existing data into the model (Krueger & Tian, 2004). We added all main effects and interactions in the model as fixed effects. In addition, following Singmann and Kellen (2017), we added a random intercept for participant and random slopes for all main effects and interactions except for the random slope for the highest-order interaction which is perfectly confounded with the residual error term and is non-identifiable. We specified “variance components” as the covariance structure to prevent correlations among random effects which otherwise led to convergence problems. The *F* and *p* values for fixed effects are reported. The partial eta squared (*η_p_^2^*) is also reported as the effect size for main effects and interactions (Richardson, 2011, Tippey and Longnecker, 2016).

#### 2.1.6 Computational modeling

We developed a hierarchical Bayesian model to explain how participants decided to ask for help from the computer in the free-choice test. We assumed that before deciding whether to ask for help, participants first monitor their memory strength for the target word in the current trial, and estimate the probability of recalling the target word, *P_rec_*. For simplicity, we assume that the subjective recall probability is the same as the objective recall probability, i.e. participants have good metacognitive sensitivity at retrieval (Rhodes & Tauber, 2011). *P_rec_* ranges between 0 and 1 and is distributed as a beta distribution:

$$P_{\mathrm{rec}}\;\sim \;Beta\left(a_{\mathrm{rec}},b_{\mathrm{rec}}\right)$$

in which *a_rec_* and *b_rec_* are the two parameters of the beta distribution for each participant. We re-parameterise the *P_rec_* distribution as follows:

$$a_{\mathrm{rec}}=U_{\mathrm{rec}}V_{\mathrm{rec}}$$

$$b_{\mathrm{rec}}=\left(1-U_{\mathrm{rec}}\right)V_{\mathrm{rec}}$$

in which *U_rec_* is the mean and *V_rec_* is approximately equivalent to the precision of the beta distribution. We specified *U_rec_* as the recall performance (the proportion of recalled trials) in the forced-recall test for each participant, which represents the overall memory strength without the help of any hint.

We assume that participants decide whether to ask for help from the computer based on the relative costs and benefits of doing so. The expected values of recalling the target with and without a hint are:

$$E\left(without\;hint\right)=P_{\mathrm{rec}}\cdot 20-\left(1-P_{\mathrm{rec}}\right)\cdot 20$$

$$E\left(with\;hint\right)=\left(P_{\mathrm{rec}}+P_{hint\_s}\right)\cdot 20-\left(1-P_{\mathrm{rec}}-P_{hint\_s}\right)\cdot 20-3$$

*P_hint_s_* denotes participants’ subjective belief about how much the hint will help them improve their performance in the memory test. The difference between the two expected values is:

$$E\left(with\;hint\right)-E\left(without\;hint\right)=P_{hint\_s}\cdot 40-3$$

This equation tells us that participants should choose to ask for help when the expected value of using the hint is higher than that for recall without hint, that is, when *P_hint_s_* > 0.075. We use a Bernoulli distribution to link participants’ choice data (1 when they choose to ask for help and 0 when they choose to answer by themselves) and model prediction for each trial in the free-choice test:

$$\mathrm{Choice}\;\sim \;\left\{\begin{array}{c} Bernoulli\left(0.999\right)\;if\;P_{hint\_s}>0.075\\ Bernoulli\left(0.001\right)\;if\;P_{hint\_s}<0.075 \end{array}\right)$$

We set the parameters in the Bernoulli distribution as 0.999 or 0.001 instead of 1 or 0 to avoid error in Bayesian sampling.

There are several possible relationships between participants’ evaluation of their memory strength and their belief about how much hints can boost their performance. First, participants may believe that the boost from a hint is independent of their own memory ability. Alternatively, they may believe that the hint can help them more when the recall probability is higher. A third possibility is that they believe the hint is more useful when it is more difficult to recall by themselves. Starting from these assumptions, we compared three different families of models of how *P_hint_s_* varies with memory strength. The Constant model assumes that *P_hint_s_* is unrelated to *P_rec_* (a constant, *β_0_*), while the Positive-slope and Negative-slope models incorporate a linear relationship between *P_hint_s_* and *P_rec_*:

$$P_{hint\_s}=\beta_{0}+\beta_{1}\cdot P_{\mathrm{rec}}$$

The Positive-slope model assumes that *β_1_* is positive (such that hints are believed to provide more benefit when memory strength is higher), while the Negative-slope model assumes that *β_1_* is negative (such that hints are believed to provide more benefit when memory strength is lower). In addition, both models assume that *P_hint_s_* falls within the 0–1 range because the hint should not negatively affect memory performance, and the improvement of memory performance given by the hint should not exceed the maximum recall probability of 1. Thus, in both models, *P_hint_s_* is 1 when *β_0_* + *β_1_* · *P_rec_* > 1, and 0 when *β_0_* + *β_1_* · *P_rec_* < 0.

During model development, we realized that we could not estimate both *β_0_* and *β_1_* in the Positive-slope and Negative-slope models. Both models assume that participants ask for help when *β_0_* + *β_1_* · *P_rec_* > 0.075, which is equal to *P_rec_* > (0.075 − *β_0_*)/*β_1_* in the Positive-slope model and *P_rec_* < (0.075 − *β_0_*)/*β_1_* in the Negative-slope model. In other words, changes in *β_0_* can be mimicked by changes in *β_1_*. We therefore defined a new parameter *C*:

$$C=\frac{0.075-\beta_{0}}{\beta_{1}}$$

*C* can be interpreted as a criterion on *P_rec_*: in the Positive-slope model, participants choose to ask for help when *P_rec_* is higher than the criterion, while in the Negative-slope model, participants ask for help when *P_rec_* is lower than the criterion.

Whether participants receive a hint for a trial in the free-choice test depends both on their choice and whether this trial has been saved. The probability of recalling the target word with the help of hint is:

$$P_{rec\_with\_hint}=\left\{\begin{array}{cc} P_{\mathrm{rec}}+P_{hint\_o} & \mathrm{if}\;P_{\mathrm{rec}}+P_{hint\_o}<1\\ 0.999 & \mathrm{if}\;P_{\mathrm{rec}}+P_{hint\_o}\geq 1 \end{array}\right)$$

Here, *P_hint_o_* is the objective boost in recall success given the hint (which is potentially different from the subjective belief in the boost in success, *P_hint_s_*). We again added a constraint in the models such that *P_rec_with_hint_* could not exceed the maximum recall probability of 1 (if *P_rec_* + *P_hint_o_* was equal to or higher than 1, *P_rec_with_hint_* was set to 0.999 to avoid error in Bayesian sampling). When the hint is not available, the recall probability for the target word is simply *P_rec_*. We used a Bernoulli distribution to link participants’ recall data (1 for successful recall and 0 for unsuccessful recall) and model prediction for each trial in the free-choice test:

$$\mathrm{Recall}\;\sim \;\left\{\begin{array}{cc} Bernoulli\left(P_{rec\_with\_hint}\right) & \mathrm{if}\;hint\;is\;avaliable\\ Bernoulli\left(P_{\mathrm{rec}}\right) & \mathrm{if}\;hint\;is\;unavaliable \end{array}\right)$$

We fitted the three models (the Constant model, Positive-slope model and Negative-slope model) to participants’ decisions about whether to ask for help and their memory performance in each trial of the free-choice test. We used Markov chain Monte Carlo (MCMC) methods implemented in JAGS (using the MATLAB interface matjags) to sample from posterior distributions of parameters (Plummer, 2003). The parameters in the models are *β_0_* (in the Constant model) or *C* (in the Positive-slope and Negative-slope model), *V_rec_* and *P_hint_o_* (see the Supplemental Information for the prior distribution of the parameters). We separately estimated parameters for each condition of our 2 (easy vs. difficult) × 2 (saved vs. unsaved) design at both the participant- and group-level. To further examine the relationship between the subjective belief and objective effect of the hints on memory performance in different experimental conditions, we also fitted different models in which we forced *β_0_* (or *C*) or *P_hint_o_* to be the same across different conditions for each participant and then compared the fit of each model. This factorial model comparison resulted in four hypothetical relationships between *β_0_* (or *C*) across different conditions: (1) easy = difficult, saved = unsaved; (2) easy ≠ difficult, saved = unsaved; (3) easy = difficult, saved ≠ unsaved; (4) easy ≠ difficult, saved ≠ unsaved. There were two different relationships between *P_hint_o_* in different conditions: (1) easy = difficult, (2) easy ≠ difficult, because participants can only receive a hint when the word pair was saved. Thus, we fitted 3 × 4 × 2 = 24 models in total.

We fitted each model with 4 chains and each chain contained 100,000 samples. We discarded 50,000 samples per chain for burn-in, resulting in 200,000 stored samples in total. Gelman and Rubin’s potential scale reduction factor R̂ was calculated for all parameters in each model (Gelman & Rubin, 1992). For 21 of the 24 models, R̂ values were <1.1 for all parameters, indicating good convergence. Models with R̂ > 1.1 were excluded from further analysis. We compared the Deviance Information Criteria (DIC) of the converged models (Spiegelhalter, Best, Carlin, & Van Der Linde, 2002), and results from the model with the lowest DIC are reported. We compared the prediction of the winning model for participants’ decision and memory performance in the free-choice test to the empirical data (a posterior predictive check). We also investigated the difference in the parameters of the winning model between different experimental conditions by comparing the posterior distribution of the parameters at the group level.

### 2.2 Results

#### 2.2.1 Predictors of offloading during learning

The proportion of saved word pairs during learning was significantly higher for difficult (*M* = 0.75, *SD* = 0.18) compared to easy pairs (*M* = 0.15, *SD* = 0.19), *t* (26) = 9.17, *p* < .001, *d* = 1.76, showing that participants’ decisions to offload were appropriately sensitive to item difficulty.

#### 2.2.2 Predictors of asking for help at test

**Fig. 2:**
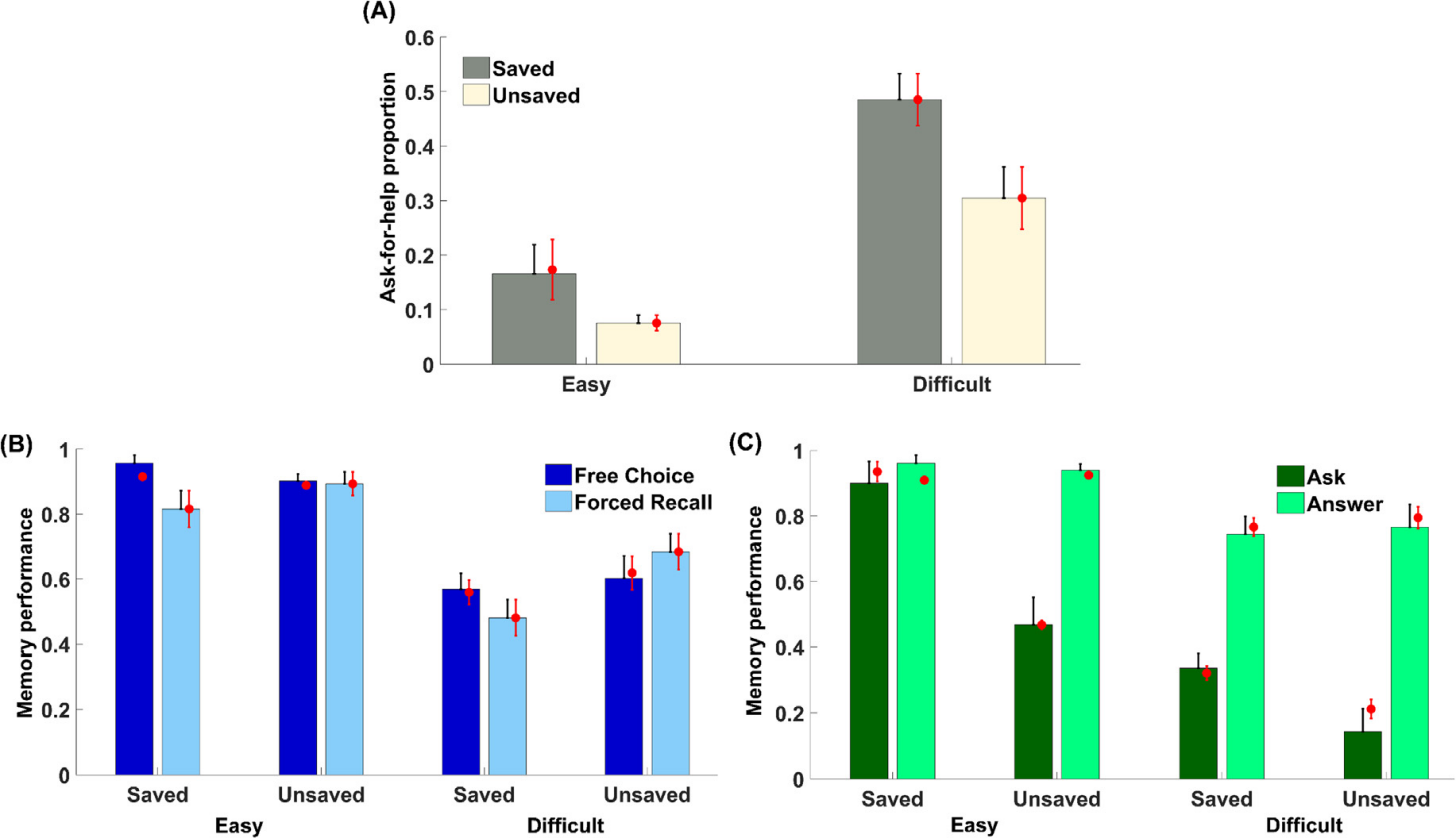
Ask-for-help behaviour and memory performance in the memory test of Experiment 1. (A) Proportion of ask-for-help trials in the free-choice test was affected by both item difficulty and whether the pair was saved. (B) Memory performance was significantly higher in the free-choice than forced-recall test for saved pairs but not for unsaved pairs. (C) Memory performance in the free-choice test was significantly higher for saved (vs. unsaved) pairs only when participants asked for help. The red points represent posterior predictives from the best-fitting computational model. Error bars represent standard errors. (For interpretation of the references to colour in this figure legend, the reader is referred to the web version of this article.)

We used a linear mixed effects model (see Methods) to evaluate predictors of asking for help during the test phase (see Fig. 2A). Participants asked for help more frequently for difficult pairs than for easy pairs, *F* (1, 24.87) = 53.18, *p* < .001, *η_p_^2^* = 0.68, and the proportion of ask-for-help trials was also higher when the word pair was saved during learning, *F* (1, 51.35) = 16.55, *p* < .001, *η_p_^2^* = 0.24, demonstrating adaptive use of offloaded information. The interaction between these predictors was not significant, *F* (1, 51.35) = 1.66, *p* = .203, *η_p_^2^* = 0.03.

#### 2.2.3 Impact of offloading on recall performance

We next used a linear mixed effects model to evaluate predictors of recall performance (see Fig. 2B). Overall, participants recalled more easy pairs than difficult pairs, *F* (1, 25.83) = 53.26, *p* < .001, *η_p_^2^* = 0.67, as expected. We also found a significant interaction between test type and whether word pairs were saved during learning, *F* (1, 42.35) = 10.88, *p* = .002, *η_p_^2^* = 0.20. Specifically, recall performance was significantly higher on free-choice compared to forced-recall trials for the saved pairs, *F* (1, 27.08) = 13.34, *p* = .001, *η_p_^2^* = 0.33, but not for the unsaved pairs, *F* (1, 26) = 1.20, *p* = .283, *η_p_^2^* = 0.04, indicating that using offloaded information significantly benefited recall performance in the free-choice test. In addition, participants recalled more unsaved than saved pairs in the forced-recall test, *F* (1, 25.04) = 7.82, *p* = .010, *η_p_^2^* = 0.24, suggesting that memory strength was overall greater for unsaved than saved pairs. This difference was not significant in the free-choice test, *F* (1, 23.21) = 0.09, *p* = .773, *η_p_^2^* < 0.01. Together this pattern of results suggests that using offloaded information, when available, improves performance by compensating for pre-existing differences in memory strength between items.

We also evaluated predictors of memory performance on free-choice trials as a function of whether subjects chose to ask for help (see Fig. 2C). Recall performance was significantly higher when participants chose to answer by themselves than to ask for help, *F* (1, 26.57) = 81.96, *p* < .001, *η_p_^2^* = 0.76, indicating that they asked for help when their own memory strength was weak. There was also a significant interaction between the effect of asking for help and whether the word pair was initially saved, *F* (1, 35.73) = 17.16, *p* < .001, *η_p_^2^* = 0.32. While memory performance did not differ for saved and unsaved word pairs when participants chose to answer by themselves, *F* (1, 26.86) < 0.01, *p* = .927, *η_p_^2^* < 0.01, they recalled more saved pairs when they asked for help, *F* (1, 22.95) = 18.24, *p* < .001, *η_p_^2^* = 0.44, suggesting a selective benefit of using offloaded information at test. Taken together, our results indicate that participants adaptively chose to use hints to boost their performance in the free-choice test.

#### 2.2.4 Computational modeling

**Fig. 3:**
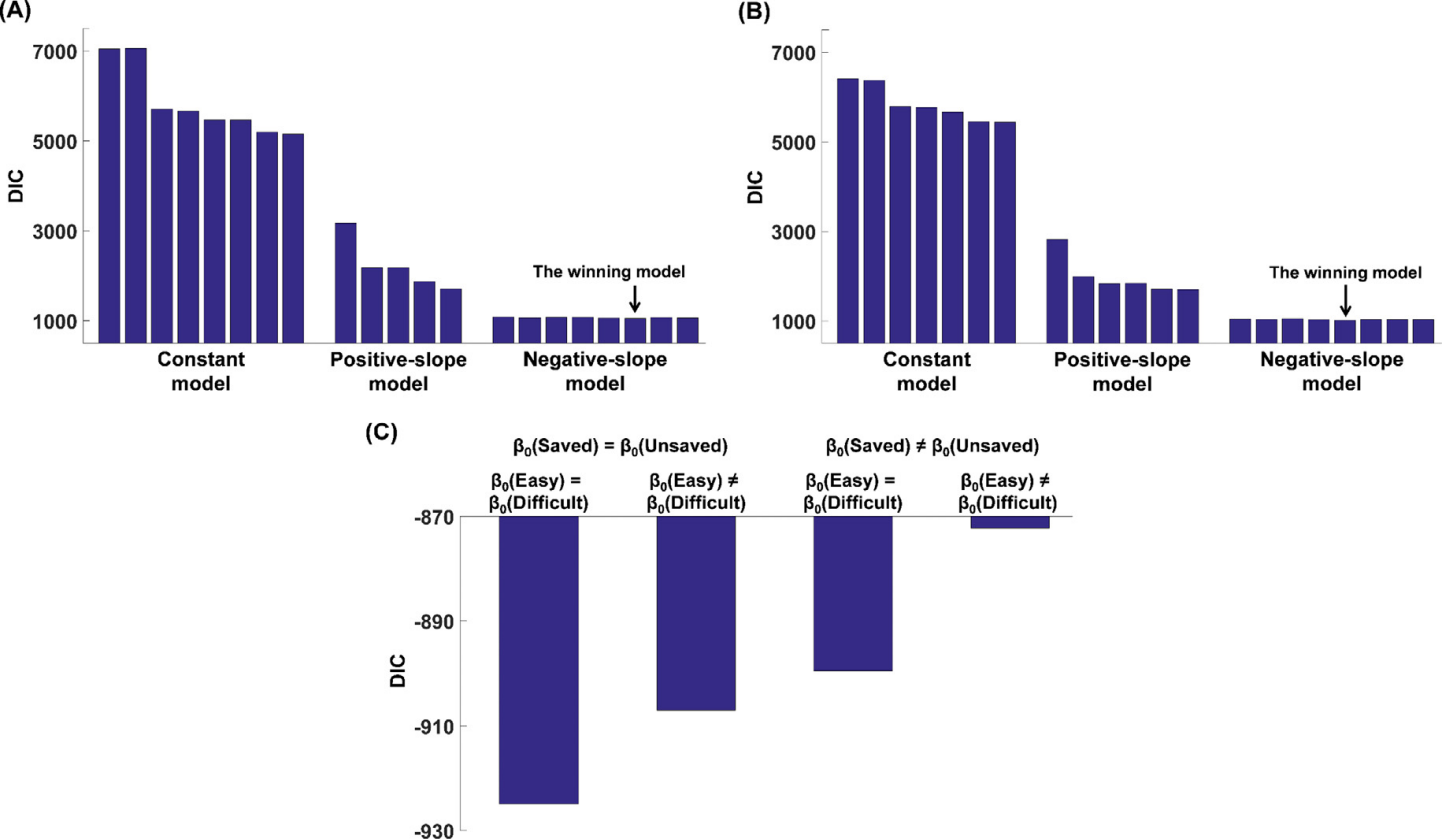
The DIC scores of alternative models (lower is better) of the data from: (A) recall performance and decisions to ask for help in Experiment 1; (B) recall performance and decisions to ask for help in Experiment 2a; (C) confidence ratings in Experiment 2a.

We fitted three sets of models (the Constant model, Positive-slope model and Negative-slope model; see Methods) to both recall performance and participants’ choices about whether to ask for help in the free-choice test. All models assume that participants’ decisions to ask for help are based on beliefs about the effects of hints on recall performance. However, the three sets of models differ in their assumptions about how these beliefs relate to metacognitive evaluations of memory performance. The DIC (a measure of model fit, where lower = better) for the converged models is shown in Fig. 3A. The winning model with the lowest DIC featured a negative correlation between beliefs about how much hints will help at test and evaluations of memory strength. In other words, when I am confident in being able to answer the question, I think a hint will be of little benefit, whereas when I am less confident I begin to recognize the value of a hint. Notably the family of Negative-slope models fitted better than a corresponding family of Constant-slope models, in which the benefit of a hint is unrelated to memory evaluation.

The winning model was able to capture both participants’ offloading behaviour and recall performance (Fig. 2). We found that the criterion for asking for help (*C*) was significantly more liberal for easy than difficult trials, mean of the difference = 0.186, 95% CI [0.070 0.295] (see Table 1). We also investigated the difference in the objective effect of the hints on recall performance (*P_hint_o_*) between easy and difficult trials, finding that the hint boosted memory performance more in the easy condition, mean of the difference = 0.487, 95% CI [0.189 0.766] (see Table 1). These results suggest participants were not only more willing to ask for help for easy than difficult pairs, but that they also accrue more performance benefit from the hint when they do so.

**Table 1:** Parameter estimates from the winning models in Experiments 1–3.

	Experiments			
	1	2a	2b	3
**Model for recall and choices**				
***C***				
Easy	0.611 (0.039)	0.649 (0.047)	0.695 (0.031)	0.620 (0.035)
Difficult	0.425 (0.042)	0.415 (0.053)	0.521 (0.046)	0.620 (0.035)
***P_hint_o_***				
Easy	0.649 (0.144)	0.224 (0.036)	0.631 (0.128)	0.501 (0.052)
Difficult	0.162 (0.033)	0.224 (0.036)	0.291 (0.035)	0.195 (0.031)

**Model for confidence ratings**				
***β_0_***				
Easy		0.291 (0.097)	0.243 (0.085)	0.221 (0.078)
Difficult		0.291 (0.097)	0.243 (0.085)	0.277 (0.049)
***β_1_***				
Easy		−0.450 (0.384)	−0.362 (0.380)	−0.413 (0.423)
Difficult		−4.396 (18.402)	−0.597 (0.629)	−0.398 (0.378)

*Note.* Standard deviations are reported in parentheses. The parameters *C*, *β_0_* and *β_1_* are constrained to be equal for saved and unsaved trials. The following parameters are constrained to be equal for easy and difficult trials in some of the experiments: *C* (Experiment 3), *P_hint_o_* (Experiment 2a) and *β_0_* (Experiments 2a and 2b).

### 2.3 Discussion

The results of Experiment 1 revealed that participants' decisions to offload both during learning and at test were affected by item difficulty, suggesting that metacognitive evaluation of difficulty may be related to the use of cognitive offloading during encoding and retrieval. In addition, we found that hints significantly improved recall performance when participants asked for help in the free-choice test, suggesting that use of offloaded information efficiently enhanced test performance.

Our best-fitting computational model indicated that participants' beliefs about whether hints could improve their performance were negatively related to evaluations of memory strength (as inferred from performance fluctuations), such that asking for help was more frequent when memory strength was weaker. Interestingly, the criterion for asking for help was more liberal for easy than difficult pairs. This result suggests that participants were more willing to ask for help for easy pairs when internal memory strength was matched, despite the actual proportion of ask-for-help trials being higher for difficult than easy pairs because memory strength was on average weaker for difficult pairs. One explanation of this criterion difference is that additional influences may lead participants to believe that hints will boost their performance more for easy than difficult pairs, even when memory strength is matched. We could not directly investigate this hypothesis in Experiment 1 because we did not have access to participants’ subjective beliefs about the effects of hints on memory performance.

The results from Experiment 1 suggest a close relationship between metacognition and cognitive offloading. In Experiment 2 we set out to directly probe metacognitive evaluations of performance by asking for confidence ratings about the ability to recall the target words. In different trials, participants were asked to rate their confidence before the memory test about being able to recall with or without the help of a hint (similar to a delayed judgment of learning (JOL) (Nelson and Narens, 1990, Rhodes and Tauber, 2011)). By comparing confidence levels between these trial types we were able to directly assay beliefs about how hints would boost performance, and quantify possible influences (such as word pair difficulty) on such beliefs. We also extended our computational modeling framework to account for participants’ confidence ratings, providing a unified account of how metacognitive evaluations of performance relate to changes in offloading behaviour.

## 3 Experiments 2a and 2b

### 3.1 Methods

#### 3.1.1 Participants

Twenty-seven participants (9 men; age: M = 24.26 years, SD = 9.68) took part in Experiment 2a at the Wellcome Centre for Human Neuroimaging, University College London. Forty participants (22 men; age: M = 34.78 years, SD = 12.47) were recruited online in Experiment 2b via the Prolific website (https://prolific.ac/). The aim of Experiment 2b was to replicate the results in Experiment 2a with an online participant pool, which is more representative compared with traditional lab-based research (Woods, Velasco, Levitan, Wan, & Spence, 2015). Participants received monetary compensation for their participation (£7.5 in Experiment 2a and £7 in Experiment 2b) and also a bonus (up to £2, dependent on performance in the memory test). Participants were tested individually, and all provided informed consent. All participants spoke English as a first language and reported normal or corrected-to-normal vision. All procedures were approved by the local ethics committee.

#### 3.1.2 Materials

The study materials were the same as those used in Experiment 1.

#### 3.1.3 Procedure

**Fig. 4:**
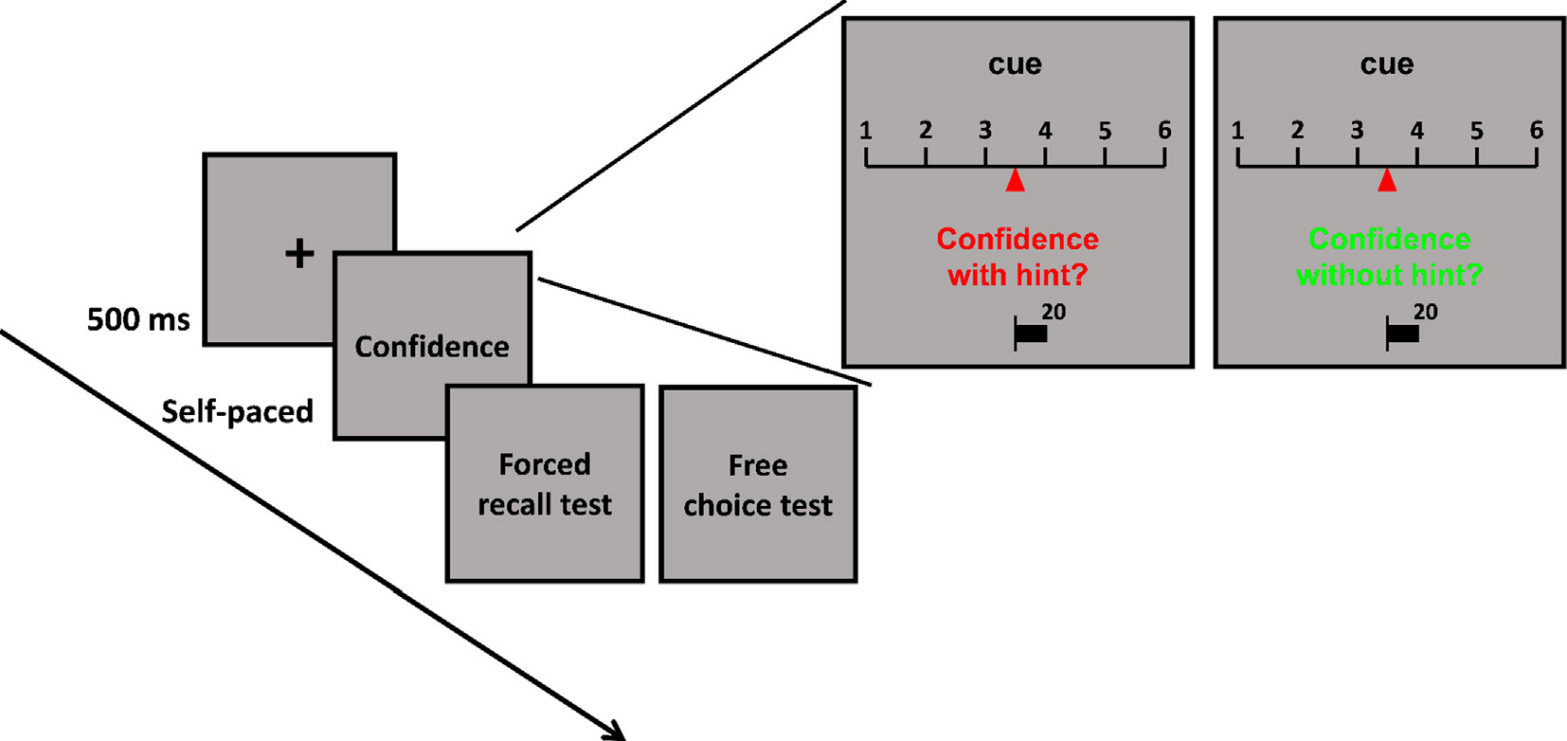
Experimental procedure of the confidence rating task in Experiment 2. In Experiment 2b, we used a 0–100 rating scale rather than the 1–6 scale.

Experiment 2a was programmed using Psychtoolbox running in MATLAB and Experiment 2b was programmed using Gorilla (Anwyl-Irvine, Massonnié, Flitton, Kirkham, & Evershed, 2018). The procedure in Experiments 2a and 2b was similar to that in Experiment 1. The only difference was that during the memory test, the cue word was presented after the fixation and participants were asked to give a confidence rating in being able to recall the word pair (see Fig. 4). All of the trials in the memory test were randomly divided into confidence-with-hint and confidence-without-hint condition. In the confidence-with-hint condition, the phrase “confidence with hint” was presented on the screen, and participants gave their confidence about being able to recall the target word with a hint. In the confidence-without-hint condition, the phrase “confidence without hint” was presented and participants gave their confidence about being able to recall the target word without help.

The two phrases were presented in different colors to remind participants of the two experimental conditions. For half of the participants the “confidence with hint” was presented in red while the “confidence without hint” was presented in green, and vice versa for the other half of the participants. Participants indicated their confidence on a sliding scale. In Experiment 2a, arbitrary scale values of 1–6 were marked at equal spacings. The initial cursor position on each trial was randomly jittered around the midpoint of the scale (±12% of scale length). Participants used the left or right arrow key to move the cursor up or down the scale. The final cursor position was recorded as a continuous variable on each trial (see Fig. 4). In Experiment 2b, a 0–100 scale was used, and the numbers 0 and 100 were marked at the two ends of the scale. Participants gave confidence ratings by dragging a circle on the slider with mouse. After giving the confidence rating, participants took the forced-recall or free-choice test in each trial as in Experiment 1.

#### 3.1.4 Design

Experiments 2a and 2b used a within-participant design. For the dependent variables that remained the same as in Experiment 1, including the proportion of saved trials during learning, recall performance and ask-for-help proportion in memory test, the experimental design was the same as in Experiment 1. An additional dependent variable in Experiments 2a and 2b was the confidence rating in the free-choice test, which was analysed as a function of item difficulty (easy vs. difficult), whether the word pair was saved during learning (saved vs. unsaved), whether participants asked for help in the free-choice test (ask-for-help vs. answer-by-themselves), and confidence type (with-hint vs. without-hint). We did not analyse the confidence ratings in the forced-recall test.

#### 3.1.5 Data analysis

Data from three blocks were collapsed in the analysis. As in Experiment 1, we used t-tests to analyse the effect of item difficulty on decisions to save the word pairs during learning and built linear mixed effect models to analyse the effect of item difficulty, whether the word pair was saved and test type (forced-recall/free-choice) on the decisions to ask for help and recall performance in the memory test. Confidence ratings were converted into a percentage scale. We were unable to conduct a full factorial 2 (easy vs. difficult) × 2 (saved vs. unsaved) × 2 (ask-for-help vs. answer-by-themselves) × 2 (confidence-with-hint vs. confidence-without-hint) analysis on confidence ratings in the free-choice test due to a high proportion of missing cells. Instead, we combined saved and unsaved trials and built linear mixed effect models to analyse the effects of item difficulty, answer/ask and confidence with/without hint on confidence ratings. We also combined easy and difficult trials and analysed the effects of saved/unsaved, answer/ask and confidence with/without hint on confidence. In addition, to investigate individual differences in the decision to ask for help and its relationship with confidence rating and memory performance, we analysed the correlation between participants’ overall proportion of ask-for-help trials in the free-choice test, mean confidence rating in the free-choice test, and overall recall performance in the forced-recall test. We also performed a mediation analysis using the PROCESS macro for SPSS (Hayes, 2013) to examine the role of confidence in mediating the effect of memory strength on decisions to ask for help.

#### 3.1.6 Computational modeling

We first fitted the 24 models from Experiment 1 to participants’ choices and memory performance in the free-choice test. For 21 of the 24 models, the R̂ values were <1.1 for all parameters, indicating good convergence. The models with R̂ > 1.1 were excluded from analysis. We then compared the DIC of the converged models. To foreshadow, the winning model in both Experiments 2a and 2b belonged to the family of Negative-slope models. For each participant, we then extracted the posterior mean of the recall probability *P_rec_* for each trial and the criterion parameter *C* for each experimental condition from the winning model, and built a new model to explain participants’ confidence ratings in the free-choice test.

We assume that when participants estimate their confidence in recalling a target word without a hint, their estimation is simply the *P_rec_* for this word pair. In contrast, when they estimate their confidence with a hint, their estimation is the sum of *P_rec_* and their belief about how much the hint could boost their performance for this pair (*P_hint_s_*). According to the assumption of the Negative-slope model, the relationship between *P_rec_* and *P_hint_s_* is:

$$P_{hint\_s}=\beta_{0}+\beta_{1}\cdot P_{\mathrm{rec}}$$

in which *β_1_* is lower than 0, and:

$$C=\frac{0.075-\beta_{0}}{\beta_{1}}$$

in which *C* is extracted from the winning model fitted to participants’ choices and memory performance in the free-choice test. This relationship places a constraint on *β_0_*: because *β_1_* is lower than 0, and *C* is a criterion on *P_rec_* about whether to ask for help and should be within the 0–1 range, *β_0_* should be higher than 0.075 to satisfy these constraints. Furthermore, *β_0_* should also be lower than 1 because *β_0_* is equal to *P_hint_s_* when *P_rec_* is 0, and should fall within the 0–1 range.

After estimating an internal probability of recalling the target word with or without a hint, participants then need to map this probability onto the confidence scale. We assume that this mapping may be biased such that:

$$\mathrm{Predicted}\;confidence\;\left(without\;hint\right)=P_{\mathrm{rec}}+bias$$

$$\mathrm{Predicted}\;confidence\;\left(with\;hint\right)=P_{\mathrm{rec}}+P_{hint\_s}+bias$$

with the constraint that the predicted confidence must be within the 0–1 range. Thus, the predicted confidence is 1 when *P_rec_* + *bias* > 1 or *P_rec_* + *P_hint_s_* + *bias* > 1, and 0 when *P_rec_* + *bias* < 0 or *P_rec_* + *P_hint_s_* + *bias* < 0.

Finally, we assume that the empirical confidence rating is drawn from a normal distribution with a mean of the predicted confidence and a standard deviation of σ_conf_, reflecting metacognitive noise in confidence reports (De Martino et al., 2013, Sanders et al., 2016):

$$\mathrm{Confidence}\;\sim \;Normal\left(Predicted\;confidence,{\sigma_{\mathrm{conf}}}^{2}\right)$$

For each experiment, we fitted the model to participants’ confidence ratings with and without hint in the free-choice test. As in Experiment 1, we used Markov chain Monte Carlo (MCMC) methods implemented in JAGS to sample from posterior distributions of parameters (Plummer, 2003). The parameters in the model are *β_0_*, *bias* and σ_conf_ (See the Supplemental Information for the prior distribution of the parameters). We separately estimated *β_0_* in 2 (easy vs. difficult) × 2 (saved vs. unsaved) conditions at both the participant- and group-level. In addition, we examined the relationship between *β_0_* in different experimental conditions. There were four different relationships between *β_0_* in different conditions for each participant: (1) easy = difficult, saved = unsaved; (2) easy ≠ difficult, saved = unsaved; (3) easy = difficult, saved ≠ unsaved; (4) easy ≠ difficult, saved ≠ unsaved. Thus, we fitted 4 models in total to the confidence data. In each experiment, we fitted each model with 4 chains and each chain contained 100,000 samples. We discarded 50,000 samples per chain for burn-in, resulting in 200,000 stored samples in total. For all of the four models in both experiments, the R̂ values were <1.1 for all parameters, indicating good convergence. We report the results from the model with the lowest DIC and compare predictions for participants’ confidence ratings to the empirical data. For each participant, we also calculated *β_1_* in each condition from *β_0_* and *C*, and compared *β_1_* across different conditions using a Wilcoxon signed rank test (we used a non-parametric test due to the presence of extreme values of *β_1_*).

### 3.2 Results

Figures of the results of Experiment 2b can be found in the Supplemental Information.

#### 3.2.1 Replication of patterns of offloading behaviour and memory performance

**Fig. 5:**
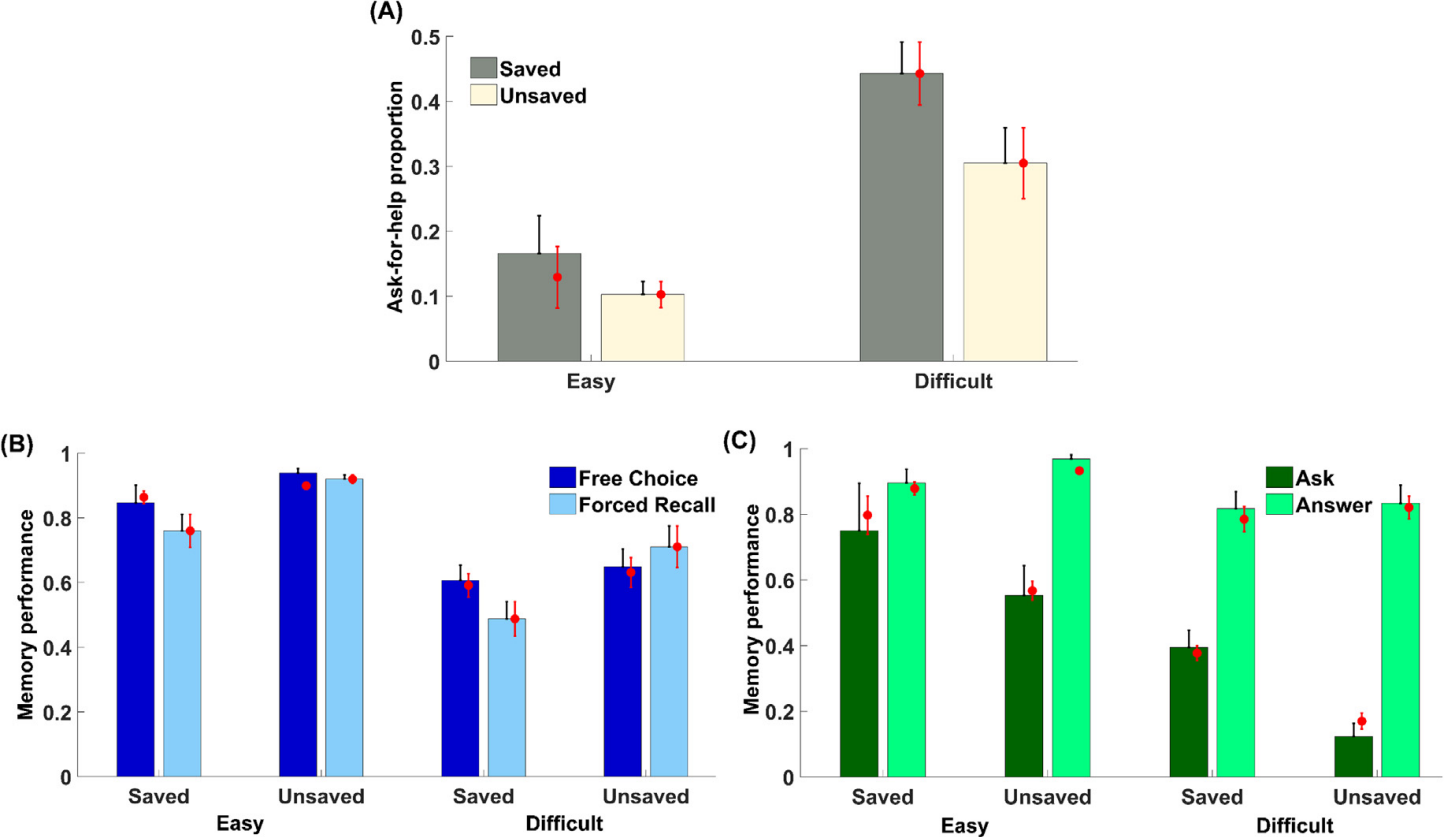
Ask-for-help behaviour and memory performance in the memory test of Experiment 2a. (A) Proportion of ask-for-help trials in the free-choice test was affected by both item difficulty and whether the pair was saved. (B) Memory performance was significantly higher in the free-choice than forced-recall test for saved pairs but not for unsaved pairs. (C) Memory performance in the free-choice test was significantly higher for saved (vs. unsaved) pairs only when participants asked for help. The red points represent posterior predictives from the best-fitting computational model. Error bars represent standard errors. (For interpretation of the references to colour in this figure legend, the reader is referred to the web version of this article.)

We first asked whether the same patterns of offloading behaviour and memory performance in Experiment 1 were observed in Experiments 2a and 2b. All key results were replicated: participants saved more word pairs in the difficult (Exp 2a: *M* = 0.77, *SD* = 0.16; Exp 2b: M = 0.71, SD = 0.21) than easy (Exp 2a: *M* = 0.12, *SD* = 0.08; Exp 2b: M = 0.08, SD = 0.13) condition, Exp 2a: *t* (26) = 18.71, *p* < .001, *d* = 3.60; Exp 2b: *t* (39) = 15.44, *p* < .001, *d* = 2.44. Recall performance for saved trials was significantly higher in the free-choice test than forced-recall test, Exp 2a: *F* (1, 44.10) = 12.34, *p* = .001, *η_p_^2^* = 0.22; Exp 2b: *F* (1, 54.76) = 13.46, *p* = .001, *η_p_^2^* = 0.20, and was significantly and selectively higher on saved (vs. unsaved) trials in the free-choice test when people asked for help, Exp 2a: *F* (1, 23.47) = 8.72, *p* = .007, *η_p_^2^* = 0.27; Exp 2b: *F* (1, 54.49) = 8.27, *p* = .006, *η_p_^2^* = 0.13. When participants answered by themselves, recall performance in the free-choice test did not differ between saved and unsaved trials in Experiment 2a, *F* (1, 43.06) = 3.79, *p* = .058, *η_p_^2^* = 0.08, but was lower for saved trials in Experiment 2b, *F* (1, 70.61) = 4.86, *p* = .031, *η_p_^2^* = 0.06. Together, these results again reveal that hints significantly improved memory performance for saved trials when participants chose to ask for help. Finally, participants again asked for help more frequently for difficult pairs compared to easy pairs, Exp 2a: *F* (1, 26.32) = 28.77, *p* < .001, *η_p_^2^* = 0.52; Exp 2b: *F* (1, 40.16) = 70.00, *p* < .001, *η_p_^2^* = 0.64, and for saved compared to unsaved pairs, Exp 2a: *F* (1, 26.58) = 5.17, *p* = .031, *η_p_^2^* = 0.16; Exp 2b: *F* (1, 76.52) = 30.54, *p* < .001, *η_p_^2^* = 0.29. In Experiment 2b there was also an interaction suggesting that the difference in ask-for-help proportion between saved and unsaved pairs was larger for difficult (vs. easy) trials, *F* (1, 76.52) = 5.64, *p* = .020, *η_p_^2^* = 0.07 (see Fig. 5 for results of Experiment 2a and Fig. S1 for Experiment 2b).

#### 3.2.2 Confidence in recall performance

**Fig. 6:**
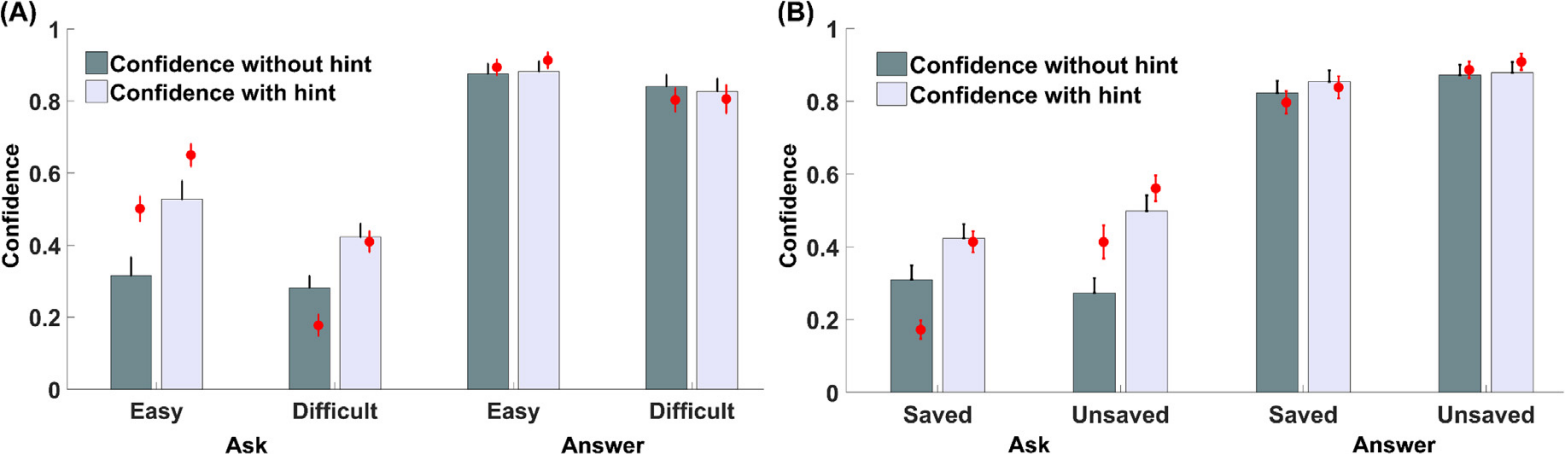
Mean confidence ratings in the free-choice test of Experiment 2a as a function of answer/ask-for-help, whether the confidence was for trials with/without a hint, and: (A) item difficulty; (B) whether the word pair was saved. Confidence was higher when participants chose to answer by themselves, and significantly modulated by the potential benefit of the hint only when participants chose to ask for help. The red points represent posterior predictives from the best-fitting computational model. Error bars represent standard errors. (For interpretation of the references to colour in this figure legend, the reader is referred to the web version of this article.)

We next turned to the confidence rating data in the free-choice test (see Fig. 6A for results of Experiment 2a and Fig. S2A for Experiment 2b). Overall, participants’ confidence was higher when they chose to answer by themselves than ask for help, Exp 2a: *F* (1, 50.21) = 106.14, *p* < .001, *η_p_^2^* = 0.68; Exp 2b: *F* (1, 64.40) = 196.38, *p* < .001, *η_p_^2^* = 0.75, suggesting that they only asked for help when their confidence about their memory was low. Their confidence in being able to provide the correct answer following a hint was also higher than without a hint, Exp 2a: *F* (1, 46.32) = 25.60, *p* < .001, *η_p_^2^* = 0.36; Exp 2b: *F* (1, 44.31) = 27.64, *p* < .001, *η_p_^2^* = 0.38, confirming that participants believed hints would boost their performance. In addition, participants’ confidence was higher for easy than difficult pairs, Exp 2a: *F* (1, 28.81) = 11.24, *p* = .002, *η_p_^2^* = 0.28; Exp 2b: *F* (1, 80.70) = 46.00, *p* < .001, *η_p_^2^* = 0.36. More importantly, we obtained a significant interaction between the potential benefit of hint and the decision to ask for help, Exp 2a: *F* (1, 46.32) = 24.44, *p* < .001, *η_p_^2^* = 0.35; Exp 2b: *F* (1, 43.36) = 17.90, *p* < .001, *η_p_^2^* = 0.29. In Experiment 2a there was also a three-way interaction between all these factors, *F* (1, 42.42) = 10.72, *p* = .002, *η_p_^2^* = 0.20.

To unpack the drivers of these interaction effects, we conducted follow-up analyses on the confidence ratings separated by whether participants chose to answer by themselves or ask for help. Participants’ confidence was significantly modulated by item difficulty regardless of their choice, Exp 2a: *F_ask_* (1, 21.08) = 4.32, *p* = .050, *η_p_^2^* = 0.17, *F_answer_* (1, 26.04) = 9.48, *p* = .005, *η_p_^2^* = 0.27; Exp 2b: *F_ask_* (1, 61.96) = 11.33, *p* = .001, *η_p_^2^* = 0.15, *F_answer_* (1, 38.65) = 57.91, *p* < .001, *η_p_^2^* = 0.60. For trials in which participants decided to ask for help, their confidence was also modulated by the potential benefit of a hint, Exp 2a: *F* (1, 23.07) = 23.58, *p* < .001, *η_p_^2^* = 0.51; Exp 2b: *F* (1, 38.95) = 21.36, *p* < .001, *η_p_^2^* = 0.35. In Experiment 2a, this effect of hints on confidence was also stronger for easy compared to difficult trials, *F* (1, 17.58) = 8.57, *p* = .009, *η_p_^2^* = 0.33. These results suggest that participants believed hints would significantly enhance their memory performance when they asked for help. In contrast, for trials in which participants decided to answer by themselves, their confidence ratings were no longer affected by the potential benefit of a hint in Experiment 2a, *F* (1, 26.23) = 0.03, *p* = .858, *η_p_^2^* < 0.01. In Experiment 2b, while confidence ratings were still modulated by the potential benefit of a hint when participants answered by themselves, *F* (1, 39.29) = 4.20, *p* = .047, *η_p_^2^* = 0.10, this effect was significantly smaller than that when they asked for help (as revealed by an interaction between ask/answer and confidence with/without hint). Overall, this pattern of ratings suggests that participants believed a hint would not be as beneficial on trials when they decided to answer by themselves.

We next asked whether the status of a word pair as saved or unsaved affected the confidence rating given in the free-choice test (see Fig. 6B for Experiment 2a and Fig. S2B for Experiment 2b). When participants decided to answer by themselves, confidence was significantly higher when the pair was unsaved compared to when it was saved, Exp 2a: *F* (1, 26.00) = 8.05, *p* = .009, *η_p_^2^* = 0.24; Exp 2b: *F* (1, 38.09) = 29.78, *p* < .001, *η_p_^2^* = 0.44. In contrast, for trials in which participants asked for help, confidence was not modulated by whether the pair was saved, Exp 2a: *F* (1, 19.57) = 1.37, *p* = .256, *η_p_^2^* = 0.07; Exp 2b: *F* (1, 69.67) = 1.62, *p* = .207, *η_p_^2^* = 0.02.

Taken together, these results are consistent with a negative correlation between participants’ evaluations of memory strength and their beliefs about how much a hint will boost performance: confidence was significantly lower when they chose to ask for help, and when they decided to answer by themselves they believed hints would provide less additional benefit.

#### 3.2.3 Computational modeling

We first fitted our three candidate sets of models (the Constant model, Positive-slope model and Negative-slope model) to participants’ recall performance and choices about whether to ask for help. The DIC scores of the converged models are shown in Fig. 3B for Experiment 2a and Fig. S3A for Experiment 2b. Replicating Experiment 1, the winning model in both Experiments 2a and 2b with the lowest DIC was drawn from the family of Negative-slope models, and the model fitted well to participants’ choice and recall performance (see Fig. 5 and Fig. S1)^1^. Finally, similar to Experiment 1, the criterion parameter *C* was significantly more liberal for easy than difficult trials, Exp 2a: mean of the difference = 0.234, 95% CI [0.090 0.367]; Exp 2b: mean of the difference = 0.174, 95% CI [0.064 0.282] (see Table 1).

We next fitted the models to participants’ confidence ratings. All models assumed a linear relationship (with negative slope) between participants’ evaluation of their own memory strength (*P_rec_*) and their belief about how much a hint would boost their performance (*P_hint_s_*). Reported confidence was modeled as a combination of these variables together with a bias parameter. Model fits were informed by memory strength (*P_rec_*) and criterion (*C*) parameters obtained from the fits of the winning model of the recall/choice data (see Methods). The winning model in both experiments was able to capture the qualitative pattern of participants’ confidence ratings (see Fig. 6 and Fig. S2). The DIC of each model is shown in Fig. 3C for Experiment 2a and Fig. S3B for Experiment 2b. In the winning model with the lowest DIC, the intercept *β_0_* in the linear relationship between *P_rec_* and *P_hint_s_* was the same across all conditions in the 2 (easy vs. difficult) × 2 (saved vs. unsaved) design (see Table 1). In contrast, *β_1_* was significantly lower for difficult (Exp 2a: *M* = −4.40, *SD* = 18.40; Exp 2b: *M* = −0.60, *SD* = 0.63) than easy (Exp 2a: *M* = −0.45, *SD* = 0.38; Exp 2b: *M* = −0.36, *SD* = 0.38) trials, Exp 2a: *Z* = 4.493, *p* < .001; Exp 2b: *Z* = 4.032, *p* < .001, again suggesting that participants believed hints could enhance their recall performance more for easy than difficult pairs even when internal memory strength was matched.

#### 3.2.4 Individual differences in offloading behaviour, confidence and memory performance

**Fig. 7:**
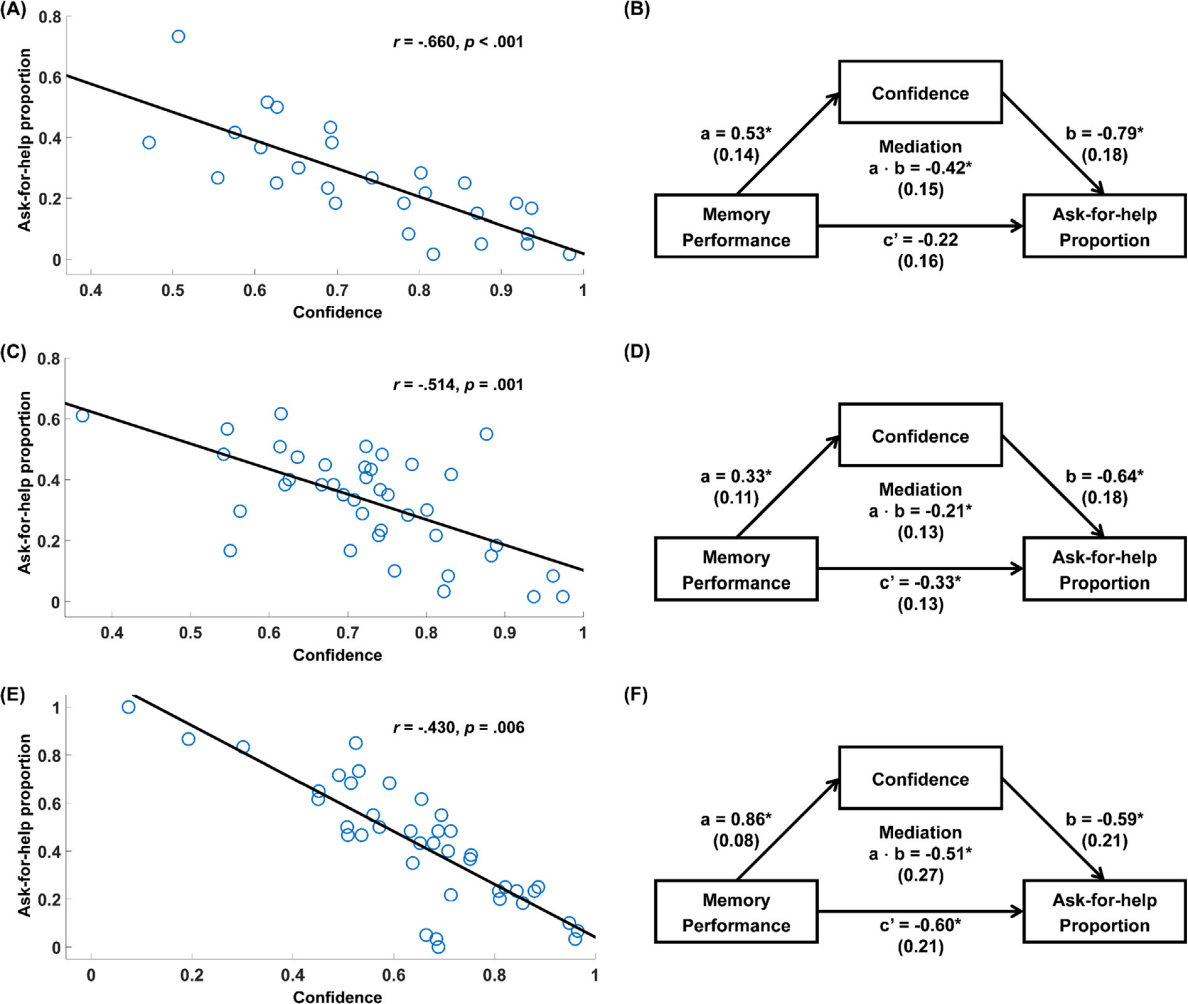
The relationships between participants’ overall proportion of ask-for-help trials in the free-choice test, mean confidence in the free-choice test, and overall memory performance in the forced-recall test in Experiments 2a, 2b and 3. There was a negative partial correlation between confidence and the ask-for-help proportion when controlling for memory performance in Experiments 2a (A), 2b (C) and 3 (E). Confidence also mediated the effect of memory performance on the ask-for-help proportion in Experiments 2a (B), 2b (D) and 3 (F). Asterisks indicate significant effects (*p* < .05). Standard errors are reported in parentheses.

Finally, we sought to characterise between-subject relationships between participants’ overall proportion of ask-for-help trials in the free-choice test, mean confidence in the free-choice test, and overall recall performance in the forced-recall test (which reflects participants’ memory strength without the help of the hints). There was a significant negative correlation between the proportion of ask-for-help decisions and mean confidence, Exp 2a: *r* = −0.782, *p* < .001; Exp 2b: *r* = −0.623, *p* < .001, revealing that participants who tended to ask for help more frequently were also less confident in their memory. The ask-for-help proportion was also negatively correlated with memory performance, Exp 2a: *r* = −0.600, *p* = .001; Exp 2b: *r* = −0.535, *p* < .001, suggesting that participants who performed worse were also more likely to take advantage of the help available. Critically, however, the negative correlation between the ask-for-help proportion and average confidence remained significant even when memory performance was controlled, Exp 2a: *r* = −0.660, *p* < .001; Exp 2b: *r* = −0.514, *p* = .001 (see Fig. 7A and C). This result suggests that metacognitive evaluation of memory – as assayed with confidence ratings – is a key driver of the decision to use offloaded information, even after accounting for differences in objective memory ability.

To formally test this hypothesis, we performed a mediation analysis to ask whether confidence mediated the effect of memory performance on the decision to ask for help (see Fig. 7B and D). Of the effect of memory performance on the ask-for-help proportion (Exp 2a: *β* = −0.64, 95% CI [−0.99, −0.29], *p* < .001; Exp 2b: *β* = −0.54, 95% CI [−0.83, −0.26], *p* < .001), 65.8% of the variance in Experiment 2a and 38.8% in Experiment 2b was accounted by confidence, resulting in a significant mediation effect (Exp 2a: *β* = −0.42, 95% CI [−0.74, −0.15]; Exp 2b: *β* = −0.21, 95% CI [−0.55, −0.03]). In addition, the direct effect of memory performance on the ask-for-help proportion was no longer significant in Experiment 2a after accounting for changes in confidence, *β* = −0.22, 95% CI [−0.55, 0.12], *p* = .194 (it remained significant in Experiment 2b, *β* = −0.33, 95% CI [−0.61, −0.06], *p* = .018). Together these results confirm that confidence is a crucial mediator between memory performance and offloading behaviour.

### 3.3 Discussion

In Experiments 2a and 2b we replicated the key relationships between participants’ offloading behaviour and memory performance obtained in Experiment 1. Participants both saved more pairs on difficult trials, and asked for help more frequently for difficult, saved pairs than easy, unsaved pairs. In turn, hints significantly enhanced memory performance when participants asked for help. By eliciting confidence ratings in Experiments 2a and 2b, we obtained direct evidence that a decision to ask for help may depend on beliefs about how much hints can boost performance. We found that people believed a hint could provide more benefit for memory performance when they chose to ask for help. In addition, beliefs about such benefits were a function of confidence in performance, as predicted by our “Negative-slope” model – when people are more confident, they believe a hint will provide less benefit. We also found that participants with lower confidence in their memory tended to ask for help more frequently even when memory performance was controlled for, and that confidence significantly mediated the effect of memory performance on the decision to ask for help. Together these results suggest that confidence (rather than memory performance) is a key factor in driving decisions to ask for help.

Importantly, the Negative-slope model was also able to capture the overall pattern of confidence rating data, providing further constraints on the functional form of the relationship between memory strength and beliefs about the helpfulness of hints. Specifically, the pattern of participants’ confidence ratings revealed a common intercept across different experimental conditions in the relationship between memory strength and hint beliefs – in other words, when confidence is very low, hints are considered uniformly beneficial. In contrast, the perceived benefit of a hint decreases more rapidly with memory strength for difficult compared to easy pairs. This feature of the model suggests that participants believe hints will boost their performance more for easy than difficult pairs even when such pairs are matched for internal memory strength. This impact of item difficulty may be due to other metamemory cues contributing to hint beliefs, as we discuss further in General Discussion.

Up until now, we have focused on the role that confidence in memory for word pairs plays in decisions to use offloaded information. However, one factor that might also affect the choice to ask for help is confidence about whether a word pair had been saved in the first place. For example, participants might be more likely to ask for help when they were sure that the current word pair had been saved. This influence was not controlled in previous experiments, because the decision about whether to save the word pair was under the participant’s control. We reasoned that variable confidence in the saved/unsaved status of the word pair might affect or confound our investigation of confidence in memory and its effect on decisions to ask for help in the free-choice test. To rule out this possibility, we next conducted Experiment 3 in which all of the word pairs were saved during learning, and hints were always available when participants asked for help in the free-choice test. In this variant of the task, participants did not need to take into account whether a pair had been saved when deciding to ask for help. We nevertheless expected that participants’ decisions to ask for help would still depend on their beliefs about how much hints could improve their memory performance, which should in turn be negatively correlated with confidence in memory.

## 4 Experiment 3

### 4.1 Methods

#### 4.1.1 Participants

Forty participants (16 men; age: M = 32.93 years, SD = 12.15) were recruited from the Prolific website (https://prolific.ac/) for monetary compensation (£6.5 plus up to £2, dependent on performance in the memory test). Participants were tested individually, and all provided online informed consent. All participants spoke English as a first language and reported normal or corrected-to-normal vision. All procedures were approved by the local ethics committee.

#### 4.1.2 Materials

The study materials were the same as those used in Experiment 1.

#### 4.1.3 Procedure

The procedure was similar to that in Experiments 2b. The only difference was that all of the word pairs were saved during learning such that participants did not need to decide whether to save each pair. Participants were told that in the free-choice test they could decide whether to use a hint to help them (i.e., the choice to ask for help), and the first two letters of the target word would be presented on the screen at the cost of 3 points whenever they decided to use the hint.

#### 4.1.4 Design and analysis

The experimental design and data analysis were similar to that in Experiments 2a and 2b. The only difference was that whether a trial was saved during learning was no longer an independent variable because there were no unsaved trials.

#### 4.1.5 Computational modeling

The model structure was the same as that in Experiments 2a and 2b. For the models fitted to participants’ choices about whether to ask for help and recall performance in the free-choice test, in each of the three model sets (Constant, Positive-slope and Negative-slope model; see Experiment 1 Methods) there were two possible relationships between the intercept *β_0_* (or *C*, the criterion for the decision to ask for help) across different conditions: (1) easy = difficult; (2) easy ≠ difficult. There were also two possible relationships between the effect of the hint on memory performance (*P_hint_o_*) in different conditions: (1) easy = difficult, (2) easy ≠ difficult. Thus, we fitted 3 × 2 × 2 = 12 models in total. For the models fitted to confidence ratings (see Experiment 2 Methods), we fitted 2 models in which the intercept *β_0_* was either the same or different across easy and difficult conditions.

### 4.2 Results

Figures of the results of Experiment 3 can be found in the Supplemental Information.

#### 4.2.1 Replication of offloading behaviour and memory performance

In Experiment 3 we again replicated the patterns of offloading behaviour observed in Experiments 1 and 2. Participants asked for help more frequently for difficult pairs compared to easy pairs, *F* (1, 39) = 76.42, *p* < .001, *η_p_^2^* = 0.66. Recall performance was significantly higher for easy than difficult pairs, *F* (1, 39) = 103.61, *p* < .001, *η_p_^2^* = 0.73, and also higher in the free-choice test than forced-recall test, *F* (1, 39) = 42.93, *p* < .001, *η_p_^2^* = 0.52, indicating that using the hints significantly benefited recall performance in the free-choice test. There was also a significant interaction, *F* (1, 39) = 6.76, *p* = .013, *η_p_^2^* = 0.15, due to the difference in memory performance between free-choice and forced-recall being higher for difficult than easy pairs (see Fig. S4).

#### 4.2.2 Replication of confidence in recall performance of the free-choice test

We also replicated the pattern of confidence ratings observed in Experiment 2, despite there now being no ambiguity about whether the pair was previously saved. We obtained a significant two-way interaction between the decision to ask for help and the potential benefit of the hint, *F* (1, 73.09) = 17.29, *p* < .001, *η_p_^2^* = 0.19. For trials in which participants decided to answer by themselves, their confidence ratings were significantly affected by item difficulty, *F* (1, 36.87) = 19.63, *p* < .001, *η_p_^2^* = 0.35, but not by the potential benefit provided by a hint, *F* (1, 69.02) = 0.59, *p* = .446, *η_p_^2^* < 0.01, suggesting that participants no longer believed a hint would help them when they decide to answer by themselves. In contrast, for trials in which participants decided to ask for help, their confidence was significantly modulated by both item difficulty, *F* (1, 24.85) = 19.91, *p* < .001, *η_p_^2^* = 0.44, and the potential benefit of the hint, *F* (1, 34.96) = 19.99, *p* < .001, *η_p_^2^* = 0.36, suggesting they believed hints could significantly boost their performance (see Fig. S5).

#### 4.2.3 Computational modeling

We again fitted our three candidate sets of models (the Constant model, Positive-slope model and Negative-slope model) to participants’ recall performance and choices about whether to ask for help. The DIC scores of the converged models are shown in Fig. S6A. Replicating previous experiments, the winning model with the lowest DIC was drawn from the family of Negative-slope models. However, unlike previous experiments, the best-fitting model was one in which the criterion for the decisions to ask for help (*C*) was constrained to be equal for easy and difficult trials (see Table 1). This model fitted well to both participants’ choice and recall performance (see Fig. S4). In addition, the hint boosted memory performance (*P_hint_o_*) to a greater extent in easy compared to difficult trials, mean of the difference = 0.306, 95% CI [0.190 0.418] (see Table 1).

We next fitted the models to participants’ confidence ratings. The winning model was able to capture the qualitative pattern of participants’ confidence ratings (see Fig. S5). The DIC of each model is shown in Fig. S6B. Although in the winning model (with the lowest DIC) the intercept *β_0_* for the linear relationship between *P_rec_* and *P_hint_s_* differed between easy and difficult trials, this difference did not reach significance when comparing parameter estimates, mean of the difference = 0.056, 95% CI [−0.126 0.223] (see Table 1). Similarly, *β_1_* did not differ between difficult (*M* = −0.40, *SD* = 0.38) and easy (*M* = −0.41, *SD* = 0.42) trials, *t* (39) = 0.267, *p* = .791, *d* = 0.04, suggesting that participants’ beliefs about how much hints could enhance their performance was similar for the two conditions.

#### 4.2.4 Individual differences in offloading behaviour, confidence and memory performance

Finally, we replicated the between-subject relationships between participants’ overall proportion of ask-for-help trials in the free-choice test, mean confidence in the free-choice test, and overall recall performance in the forced-recall test. There was a significant negative correlation between the proportion of ask-for-help decisions and mean confidence, *r* = −0.837, *p* < .001, and this correlation remained significant even when memory performance was controlled, *r* = −0.430, *p* = .006 (see Fig. 7E). As in Experiment 2, we then performed a mediation analysis to ask whether confidence formally mediated the effect of memory performance on the decision to ask for help (see Fig. 7F). Of the effect of memory performance on the ask-for-help proportion (*β* = −1.11, 95% CI [−1.35, −0.87], *p* < .001), 46.1% of the variance was accounted by confidence, resulting in a significant mediation effect (*β* = −0.51, 95% CI [−1.12, −0.11]). As in Experiment 2b, the direct effect of memory performance on the ask-for-help proportion remained significant after accounting for changes in confidence, *β* = −0.60, 95% CI [−1.02, −0.18], *p* = .006. Taken together, these results highlight confidence as a key driver of the decision to use offloaded information.

### 4.3 Discussion

In Experiment 3, we replicated the key results from Experiments 1 and 2 despite all of the word pairs now being saved during learning, thus removing any ambiguity about the saved/unsaved status of the pair during test. Participants’ decisions to ask for help were again predicted by beliefs about how much hints could boost performance, and confidence was identified as a key driver of the decision to use offloaded information. These results could also be predicted by our “Negative-slope” model, revealing that participants believed hints would provide less benefit when they had higher confidence about their own memory. However, in contrast to the previous experiments, participants’ beliefs about the benefit of the hint did not differ between easy and difficult trials. We consider potential reasons for this difference in General Discussion.

## 5 General Discussion

Although previous studies have revealed that cognitive offloading may improve memory performance (Gilbert, 2015a, Gilbert, 2015b, Risko and Dunn, 2015), few studies have investigated when and how people rely on external sources during encoding and retrieval, and whether people’s offloading behaviour is related to metacognitive evaluation. To address this question, in four experiments we asked participants to learn word pairs and then decide both whether to save each pair during encoding (in Experiments 1 and 2), and whether to ask for help at test. In Experiments 2 and 3, participants also evaluated their confidence in being able to recall the target words with or without the help of a hint. Our results suggest that the use of offloaded information at test can significantly boost memory performance, and that decisions to use offloaded information are closely related to metacognitive evaluation about expected performance across different conditions. Specifically, participants’ confidence about their own memory ability was negatively correlated with their beliefs about how much the hints could enhance their performance. Experiment 3 further supported these results even when participants did not need to consider whether each trial had been saved during learning.

A metacognitive model of cognitive offloading suggests that whether people rely on themselves or external sources in a task depends on metacognitive evaluation (Dunn and Risko, 2016, Risko and Gilbert, 2016). Previous studies reveal that the number of to-be-remembered targets can significantly affect the reliance on external sources: people’s confidence about being able to recall the targets decreases when the number of targets increases, which results in more frequent use of offloaded information (Gilbert, 2015a, Gilbert, 2015b, Redshaw et al., 2018, Risko and Dunn, 2015). Similarly, our results showed that whether participants chose to save word pairs was also influenced by item difficulty, manipulated here by the semantic relatedness between cue and target words. Many studies have shown that people’s confidence in recalling weakly-related word pairs is significantly lower than recalling strongly-related pairs (Mueller et al., 2013, Undorf and Erdfelder, 2015), which may have led them to save more weakly-related pairs during learning. A similar sensitivity to item difficulty was also found at retrieval: participants tended to ask for help more frequently for difficult than easy pairs in the free-choice test. The effect of difficulty on use of offloaded information at test remained the same in Experiment 3 even when removing ambiguity about whether each item had been saved, further confirming a close relationship between offloading decisions and metacognitive evaluations of item difficulty.

Although participants in our experiments asked for help more frequently in the free-choice test for saved pairs, they sometimes sought hints for unsaved pairs even when none was available. One possible explanation for this behaviour is that participants asked for help for these pairs out of desperation when they could not recall the target words and knew these pairs had not been saved. However, this explanation seems unlikely, because when participants do not know the answer for an unsaved word pair they are better off guessing rather than accepting a loss of 3 points. A more plausible explanation is that participants might be unsure whether these pairs had been saved when they decided to ask for help for unsaved pairs. In Experiment 3 we sought to minimize the impact of this uncertainty by ensuring all pairs were saved. Future studies may wish to apply the current paradigm to directly investigate the intriguing role of confidence in the offloading process itself.

Our results are consistent with previous studies showing that cognitive offloading is an efficient learning strategy. Cognitive offloading has been found to improve performance in short-term memory and prospective memory tasks, for both adults and children, and in both laboratory and naturalistic environments (Gilbert, 2015a, Gilbert, 2015b, Redshaw et al., 2018, Risko and Dunn, 2015). In our experiments, recall performance in the free-choice test was significantly higher when participants asked for help, due to the adaptive use of hints, suggesting that the benefit of offloading also extends to tests of long-term memory. Furthermore, we found that confidence ratings at test were also higher when participants chose to answer by themselves than ask for help, and that subjects with lower overall confidence were also more likely to ask for help. These results are consistent with a metacognitive model of offloading – people seek help when their confidence in their own ability is low (Desender et al., 2018, Goupil et al., 2016). Similar results have also been obtained in a previous survey study that showed a negative correlation between self-reported internal memory ability and use of external memory (Finley et al., 2018).

The results of Experiments 1 and 2 also revealed that in the forced-recall test (when hints were not available), memory performance was higher for unsaved compared to saved items. There are two possible explanations for this difference. First, participants might initially choose to save pairs with higher difficulty, and thus be able to recall more of the easier, unsaved pairs when hints are unavailable. A second explanation is that when participants decide to save a word pair, they put less effort into further encoding this pair due to a (often correct) belief that they will be able to ask for help in the later memory test. Thus offloading to the environment might lead to “adaptive forgetting” of offloaded information (Sparrow et al., 2011, Storm and Stone, 2015). Our experiments cannot distinguish these two possibilities, but this hypothesis could be tested in future experiments by introducing trials during encoding in which the option to offload is not allowed. In addition, we found that there was no significant difference in recall performance between the saved and unsaved trials when hints were available, suggesting the use of offloaded information was able to boost performance for saved trials and compensate for pre-existing differences in memory strength between these conditions.

In Experiments 2 and 3, participants’ confidence ratings about future performance was closely linked to their subjective beliefs about how much hints would boost performance. Participants’ confidence was significantly lower when they chose to ask for help than answer by themselves, and the best-fitting computational model indicated a negative relationship between participants’ memory confidence and beliefs about how much hints could boost their performance. Our results build on and extend previous findings showing that people tend to offload information in memory tasks when their evaluation of their own memory ability is low (Gilbert, 2015b, Risko and Dunn, 2015), and further indicate that beliefs about the benefit of offloading are themselves shaped by metacognitive confidence in performance.

The winning model for the confidence ratings in Experiment 2 revealed that a perceived benefit of hints decreases more rapidly with memory strength for difficult compared to easy pairs. In other words, participants believed that hints could boost performance to a greater extent for easy pairs even when internal memory strength was matched. Notably, because the overall ask-for-help proportion in the free-choice test was higher for difficult pairs, this pattern would have remained hidden without the insights provided by the model (Lewandowsky & Farrell, 2011). This difference in hint belief might result from a difference in the amount of retrieved information related to the target words in easy and difficult pairs. In the current study, item difficulty was manipulated by semantic relatedness. When participants decide whether to ask for help, they might first attempt to search for the target word in memory and monitor the outcome of the search. Even when retrieval of the target word fails, they might retrieve semantic or contextual information related to the target (Koriat, 1993), which would be more easily activated for strongly-related than weakly-related pairs (Grimaldi & Karpicke, 2012). The relatively large amount of information related to the unrecalled target in strongly-related pairs might in turn lead participants to believe that they would be more likely to retrieve the target words with the help of the hints (Koriat, 1993, Metcalfe et al., 2017). The consequence of this mixture of difficulty and target-accessibility effects is that participants might be overall more willing to ask for help for the pairs with medium difficulty. This pattern is also consistent with the region of proximal learning (RPL) theory of self-regulated learning (Metcalfe & Kornell, 2005), which proposes that people invest most of their time learning materials with medium difficulty. More broadly, many studies have shown that metamemory is affected by various cues including the characteristics of the study items and the conditions of learning (Bjork et al., 2013, Koriat, 1997, Rhodes, 2015). The current study reveals that people’s use of offloaded information is affected by at least one of these cues (semantic relatedness). Future studies could profitably manipulate other cues such as familiarity or fluency (Bjork et al., 2013), and investigate the effect of metacognitive illusions on cognitive offloading (e.g., Rhodes & Castel, 2008).

We also considered whether participants’ decisions to ask for help might be related to their confidence about whether word pairs had been saved during learning, rather than their confidence in the memory itself. This potential influence was not controlled in Experiments 1 and 2. In contrast, in Experiment 3 all of the word pairs were saved during learning, such that there was no ambiguity about the status of the pair during retrieval. Importantly, the key results from Experiments 1 and 2 were replicated in Experiment 3: participants’ decisions to ask for help were closely related to their beliefs about how much hints could boost their performance, which was negatively correlated with the confidence about their own memory.

However, one notable difference was that people’s beliefs about the effect of hints on performance did not differ between easy and difficult pairs in Experiment 3. We think there are at least two possible explanations for this difference. First, the act of making offloading decisions during learning in Experiments 1 and 2 might have affected the processing of semantic associations for easy word pairs. Previous research has shown that the involvement of metamemory judgments affects memory process selectively for strongly-related word pairs (Double et al., 2018, Soderstrom et al., 2015). For example, Soderstrom et al. (2015) asked participants to learn a list of strongly and weakly related pairs. Half of the participants also needed to make predictions during learning about their performance on a subsequent test. The participants who made predictions showed selectively enhanced performance for strongly-related word pairs, but not for weakly-related pairs. Here we found a similar effect of offloading decisions on memory: forced-recall performance was significantly higher in Experiment 2b (with save decisions) than Experiment 3 (without save decisions) for easy pairs (*t* = 2.821, *p* = .006), but not for difficult pairs (*t* = 0.558, *p* = .579)^2^. Thus, the involvement of save decisions might selectively foster the processing of semantic relatedness for easy pairs, and lead participants to believe that hints would provide more benefit.

A second possibility is that participants’ confidence about whether each pair had been saved might play a role in their decisions to ask for help. Our models assume that participants ask for help based on their confidence in being able to retrieve the target word, but do not consider the influence of confidence in whether help is available in the first place. For instance, if participants mistakenly believed easy pairs were more likely to be saved than difficult pairs, then this might promote a decision to ask for help even when memory confidence was equated between pairs. Future studies could measure confidence about whether each pair has been saved to test this hypothesis.

In Experiments 2 and 3, we found that participants with lower confidence tended to ask for help more frequently even when memory performance was controlled, and that confidence mediated the relationship between memory performance and the decision to ask for help. These results again suggest that metacognitive evaluation of performance (rather than changes in memory performance *per se*) is a key driver of cognitive offloading. We note however that such a dissociation between performance and confidence is not directly captured by our current computational model, which is a first-order model and assumes that confidence is tightly coupled to memory performance. In contrast, these results may be better accounted for by a second-order model which allows for differential influences on confidence and performance (Fleming & Daw, 2017). Future research may profitably extend the model developed in the current study to construct a second-order architecture that is able to fully capture the relationship between performance, confidence and offloading behaviour (Dunn & Risko, 2016).

The use of cognitive offloading during learning is increasingly ubiquitous due to the rapid development of technology (Risko & Gilbert, 2016). For example, we often set reminders using a smartphone or take notes with a laptop when listening to lectures. By understanding when and how people rely on these external tools during learning, psychologists and educators can guide people towards more effective use of modern technologies to maximise their effect on memory performance. The current research highlights a close relationship between metacognitive evaluation (confidence) about memory performance and offloading behaviour during learning.

We should also note that there are clear differences between the offloading process as studied here and everyday life. First, while participants in our experiments could only receive a two-letter hint when they asked for help for saved word pairs, people typically have access to the full information they need when retrieving from external sources such as computer files or smartphones. Another difference is that retrieving information from external sources is typically more time-consuming than relying on memory (Arango-Muñoz, 2013). This relatively low efficiency of external memory may be taken into account when people decide whether to use offloaded information (Schönpflug, 1986) – a cost we attempted to simulate in the current study through the loss of points. If there is no cost for use of external memory, people may be more likely to routinely seek help from external sources (even if they are highly confident in their internal memory) because such hints are usually beneficial. However, presumably there is always some implicit cost (e.g. the energetics of the cognitive/motor processes needed to ask for and process the hint), such that hint use may still tradeoff against confidence in those circumstances. Future research is needed to examine how our findings translate into contexts in which people are learning from more naturalistic materials (e.g., textbooks or lectures) in their daily learning environment, and whether offloading behaviours can be optimized in such scenarios.

## Declaration of Competing Interest

None.

## Data Availability

Data and analysis code are available here: https://github.com/XiaoHuPsy/HuLuoFleming.

## Footnotes

1 Unlike in Experiment 1, the best-fitting model in Experiment 2a was one in which the effect of hint on objective memory performance (*P_hint_o_*) was constrained to be equal for easy and difficult trials. In contrast, the hint boosted memory performance more in easy than difficult trials in Experiment 2b, mean of the difference = 0.340, 95% CI [0.059 0.580], replicating the findings of Experiment 1 (see Table 1).

2 We compare the recall performance in Experiments 2b and 3 because participants in these two experiments came from the same online participant pool.
