# Supplementary material for: A role for metamemory in cognitive offloading
Source: Cognition. 2019 Dec;193:104012. doi: 10.1016/j.cognition.2019.104012 (PMC6838677; doi:10.1016/j.cognition.2019.104012)
Supplement: Supplemental Information [file mmc1.docx]

**Supplemental Information**

**Prior distribution of computational model parameters**

In Experiments 1 to 3, we fitted three models (the Constant model, Positive-slope model and Negative-slope model) to participants’ decisions about whether to ask for help and their memory performance in each trial of the free-choice test. The parameters in the models are *β_0_* (in the Constant model) or *C* (in the Positive-slope and Negative-slope model), *V_rec_* and *P_hint_o_*. For the Constant model, the prior distributions were:

$$\beta_{0}\sim Beta\left( a_{\beta_{0}},b_{\beta_{0}} \right)$$

$$a_{\beta_{0}}=U_{\beta_{0}}V_{\beta_{0}}$$

$$b_{\beta_{0}}=\left( 1-U_{\beta_{0}} \right)V_{\beta_{0}}$$

$$U_{\beta_{0}}\sim Beta\left( 1,1 \right)$$

$$V_{\beta_{0}}\sim Gamma\left( 1,20 \right)$$

$$P_{hint\_o}\sim Beta\left( a_{hint\_o},b_{hint\_o} \right)$$

$$a_{hint\_o}=U_{hint\_o}V_{hint\_o}$$

$$b_{hint\_o}=\left( 1-U_{hint\_o} \right)V_{hint\_o}$$

$$U_{hint\_o}\sim Beta\left( 1,1 \right)$$

$$V_{hint\_o}\sim Gamma\left( 1,20 \right)$$

$$V_{rec}\sim HalfNormal(0,{\sigma_{v\_rec}}^{2})$$

$$\sigma_{v\_rec}\sim HalfCauchy\left( 0,5 \right)$$

The means of *β_0_* and *P_hint_o_* at the group level, *U_β0_* and *U_hint_o_*, were drawn from non-informative prior distributions Beta (1, 1). *V_β0_* and *V_hint_o_*, which roughly characterise the precision of the distributions of *β_0_* and *P_hint_o_*, were drawn from gamma distributions with shape parameters of 1 and rate parameters of 20, as suggested by Laskey (2018). The distribution of *V_rec_* was characterised by a half-normal distribution, which only contains one parameter and is useful for describing the distribution of variables bounded below by 0. The standard deviation of this half-normal distribution, *σ_v_rec_*, was drawn from a half-Cauchy distribution, widely used as a prior distribution for standard deviations (Polson & Scott, 2012).

For the Positive-slope and Negative-slope models, the prior distributions of *P_hint_o_* and *V_rec_* were the same as in the Constant model. *C* was distributed as:

$$C\sim Beta\left( a_{C},b_{C} \right)$$

$$a_{C}=U_{C}V_{C}$$

$$b_{C}=\left( 1-U_{C} \right)V_{C}$$

$$U_{C}\sim Beta\left( 1,1 \right)$$

$$V_{C}\sim Gamma\left( 1,20 \right)$$

In Experiments 2 and 3, we fitted a model to participants’ confidence ratings in the free-choice test. The parameters in the model are *β_0_*, *bias* and σ_conf_. The prior distribution of the parameters was:

$$\beta_{0}=0.925*\beta_{0\_new}+0.075$$

$$\beta_{0\_new}\sim Beta\left( a_{\beta_{0\_new}},b_{\beta_{0\_new}} \right)$$

$$a_{\beta_{0\_new}}=U_{\beta_{0\_new}}V_{\beta_{0\_new}}$$

$$b_{\beta_{0\_new}}=\left( 1-U_{\beta_{0\_new}} \right)V_{\beta_{0\_new}}$$

$$U_{\beta_{0\_new}}\sim Beta\left( 1,1 \right)$$

$$U_{\beta_{0}}=0.925*U_{\beta_{0_{new}}}+0.075$$

$$V_{\beta_{0\_new}}\sim Gamma\left( 1,20 \right)$$

$$bias=2*{bias}_{new}-1$$

$${bias}_{new}\sim Beta\left( a_{{bias}_{new}},b_{{bias}_{new}} \right)$$

$$a_{{bias}_{new}}=U_{{bias}_{new}}V_{{bias}_{new}}$$

$$b_{{bias}_{new}}=\left( 1-U_{{bias}_{new}} \right)V_{{bias}_{new}}$$

$$U_{{bias}_{new}}\sim Beta\left( 1,1 \right)$$

$$V_{{bias}_{new}}\sim Gamma\left( 1,20 \right)$$

$$\sigma_{conf}\sim HalfCauchy\left( 0,5 \right)$$

We first transformed *β_0_* (ranging from 0.075-1) into *β_0_new_* (ranging from 0-1) and put a beta prior on *β_0_new_*. The mean of *β_0_* at the group level (*U_β0_*) was then calculated based on the mean of *β_0_new_* at the group level (*U_β0_new_*). Similarly, we transformed *bias* (ranging from -1 – 1) into *bias_new_* (ranging from 0-1) and put a beta prior on *bias_new_*. A half-Cauchy prior was used for σ_conf_.

**References**

Laskey, K. B. (2018). Bayesian Inference and Decision Theory. Retrieved from http://seor.vse.gmu.edu/~klaskey/SYST664/Bayes_Unit7.pdf

Polson, N. G., & Scott, J. G. (2012). On the half-cauchy prior for a global scale parameter. *Bayesian Analysis*, *7*(4), 887–902. https://doi.org/10.1214/12-BA730

Table S1

*Word pairs used in the current study*

|  | Easy word pairs | | |  | Difficult word pairs | | |
| --- | --- | --- | --- | --- | --- | --- | --- |
|  | Cue word | Target word | Forward associative strength |  | Cue word | Target word |  |
| **Practice block** | | | | | | |  |
|  | alcohol | drink | 0.27 |  | acre | sheet |  |
|  | brass | band | 0.26 |  | demon | mail |  |
|  | burden | load | 0.27 |  | ivory | star |  |
|  | inch | mile | 0.26 |  | painter | gray |  |
|  | jacket | coat | 0.29 |  | residue | murder |  |
|  | spoke | wheel | 0.26 |  | sunset | beef |  |
| **Formal experiment** | | | | | | |  |
|  | ankle | foot | 0.28 |  | bark | mine |  |
|  | atom | bomb | 0.49 |  | bullet | smoke |  |
|  | aunt | uncle | 0.61 |  | coin | branch |  |
|  | banner | flag | 0.43 |  | cook | mirror |  |
|  | barrel | beer | 0.51 |  | costume | fruit |  |
|  | bloom | flower | 0.51 |  | cottage | smile |  |
|  | boot | shoe | 0.38 |  | crystal | chin |  |
|  | bosom | breast | 0.28 |  | damage | seed |  |
|  | boulder | rock | 0.4 |  | dirt | goal |  |
|  | bread | butter | 0.53 |  | dock | cash |  |
|  | breeze | wind | 0.52 |  | dress | card |  |
|  | brother | sister | 0.68 |  | factory | pencil |  |
|  | butcher | meat | 0.48 |  | faint | gate |  |
|  | cage | bird | 0.55 |  | flame | pound |  |
|  | cell | prison | 0.41 |  | flesh | dollar |  |
|  | cellar | wine | 0.32 |  | golf | shadow |  |
|  | circle | round | 0.31 |  | graph | beauty |  |
|  | core | apple | 0.76 |  | grip | male |  |
|  | diamond | ring | 0.34 |  | guard | suit |  |
|  | dive | swim | 0.3 |  | hammer | stem |  |
|  | dream | sleep | 0.25 |  | hunt | ocean |  |
|  | embrace | kiss | 0.33 |  | lake | dozen |  |
|  | express | train | 0.56 |  | lever | pick |  |
|  | fabric | cloth | 0.39 |  | limp | brush |  |
|  | flock | sheep | 0.76 |  | meadow | curve |  |
|  | flush | toilet | 0.27 |  | medicine | artist |  |
|  | fork | knife | 0.65 |  | neck | soil |  |
|  | heap | pile | 0.32 |  | nest | metal |  |
|  | hose | pipe | 0.47 |  | nurse | moon |  |
|  | injury | hurt | 0.31 |  | pair | dust |  |
|  | joke | laugh | 0.43 |  | palm | trip |  |
|  | judge | jury | 0.44 |  | paste | meal |  |
|  | juice | orange | 0.38 |  | planet | post |  |
|  | king | queen | 0.44 |  | plate | knee |  |
|  | lawn | grass | 0.33 |  | poll | shirt |  |
|  | leap | jump | 0.31 |  | portrait | iron |  |
|  | link | chain | 0.6 |  | powder | coast |  |
|  | pepper | salt | 0.58 |  | praise | supper |  |
|  | profit | loss | 0.35 |  | protest | camp |  |
|  | quack | duck | 0.42 |  | ribbon | crowd |  |
|  | quest | search | 0.35 |  | roof | victim |  |
|  | remedy | cure | 0.43 |  | route | adult |  |
|  | resort | holiday | 0.28 |  | salad | dance |  |
|  | roar | lion | 0.48 |  | saloon | pocket |  |
|  | sail | boat | 0.43 |  | scratch | fish |  |
|  | silver | gold | 0.59 |  | seal | tube |  |
|  | singer | song | 0.38 |  | shear | chest |  |
|  | sketch | draw | 0.31 |  | sleeve | tape |  |
|  | slope | hill | 0.3 |  | tank | birth |  |
|  | spark | plug | 0.28 |  | touch | wave |  |
|  | steam | engine | 0.28 |  | track | fence |  |
|  | sugar | sweet | 0.44 |  | travel | gift |  |
|  | thread | needle | 0.6 |  | trick | novel |  |
|  | throat | sore | 0.34 |  | tune | storm |  |
|  | timber | wood | 0.42 |  | turtle | yard |  |
|  | umbrella | rain | 0.58 |  | village | lady |  |
|  | willow | tree | 0.55 |  | vinegar | steel |  |
|  | winter | snow | 0.25 |  | whisper | coal |  |
|  | wrath | anger | 0.49 |  | wing | desk |  |
|  | wreck | ship | 0.59 |  | wound | coffee |  |

*Note*. Data for the forward associative strengths are from the Edinburgh Associative Thesaurus (Kiss, Armstrong, Milroy, & Piper, 1973). The forward associative strengths for all of the difficult pairs are 0.

Table S2

*Relationship between points and monetary bonus*

| Total points in three blocks | Bonus |
| --- | --- |
| 0 – 500 | £0.5 |
| 500 – 1000 | £1.0 |
| 1000 – 1500 | £1.5 |
| > 1500 | £2.0 |

**
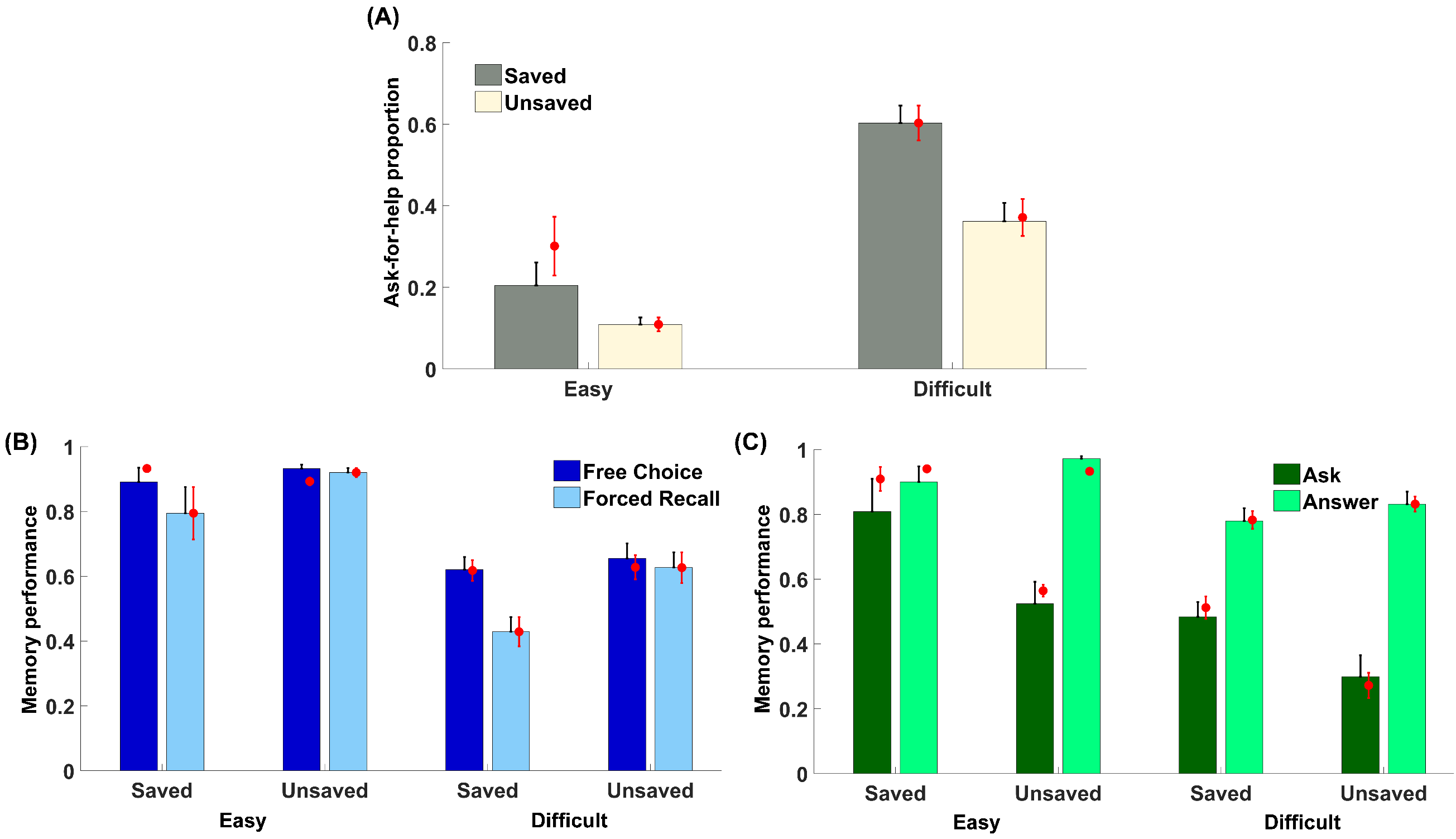
Figure S1**. Ask-for-help behaviour and memory performance in the memory test of Experiment 2b. (A) Proportion of ask-for-help trials in the free-choice test was affected by both item difficulty and whether the pair was saved. (B) Memory performance was significantly higher in the free-choice than forced-recall test for saved pairs but not for unsaved pairs. (C) Memory performance in the free-choice test was significantly higher for saved (vs. unsaved) pairs when participants asked for help, but lower for saved pairs when they answered by themselves. The red points represent posterior predictives from the best-fitting computational model. Error bars represent standard errors.

**
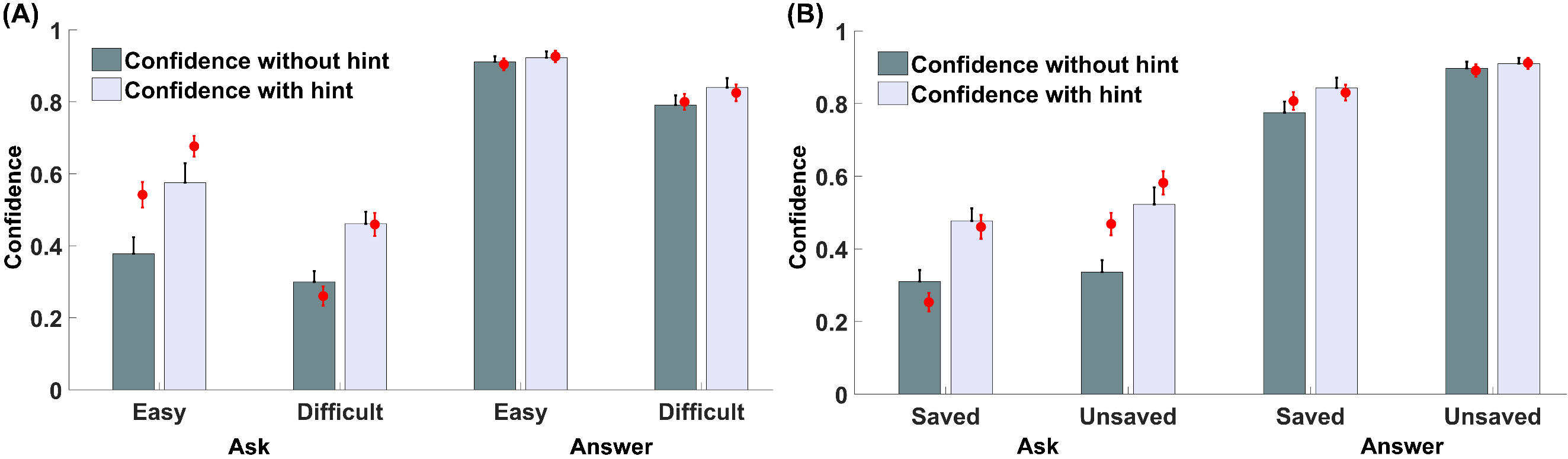
Figure S2**. Mean confidence ratings in the free-choice test of Experiment 2b as a function of answer/ask-for-help, whether the confidence was for trials with/without a hint, and: (A) item difficulty; (B) whether the word pair was saved. Participants had higher confidence when they chose to answer by themselves, and believed hints could provide larger benefit when they chose to ask for help. The red points represent posterior predictives from the best-fitting computational model. Error bars represent standard errors.

**
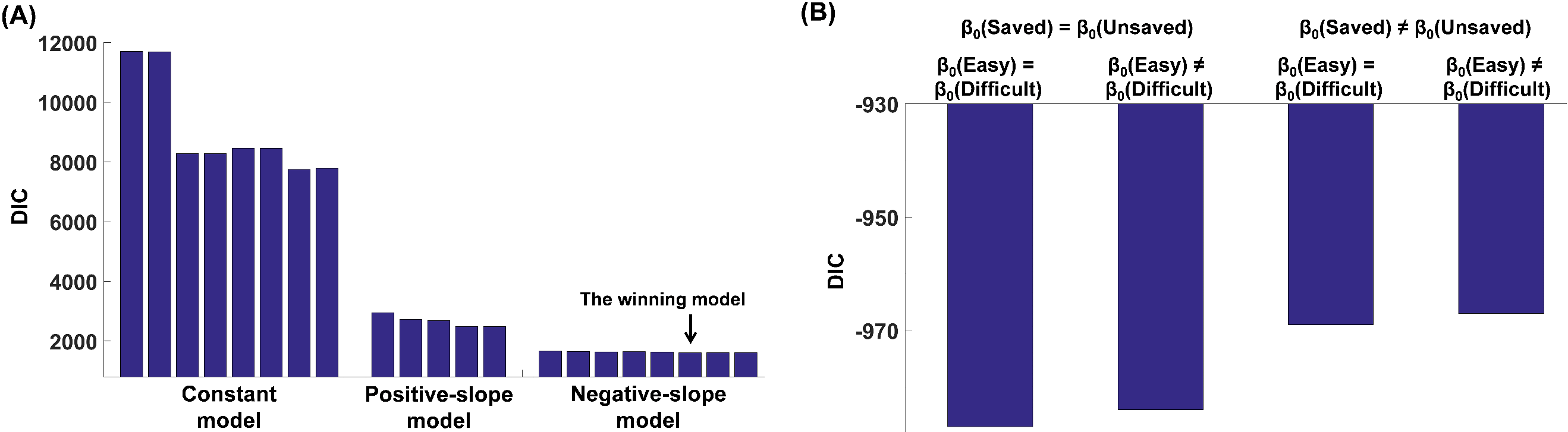
Figure S3**. The DIC scores of alternative models (lower is better) of the data from: (A) recall performance and decisions to ask for help in Experiment 2b; (B) confidence ratings in Experiment 2b.

**
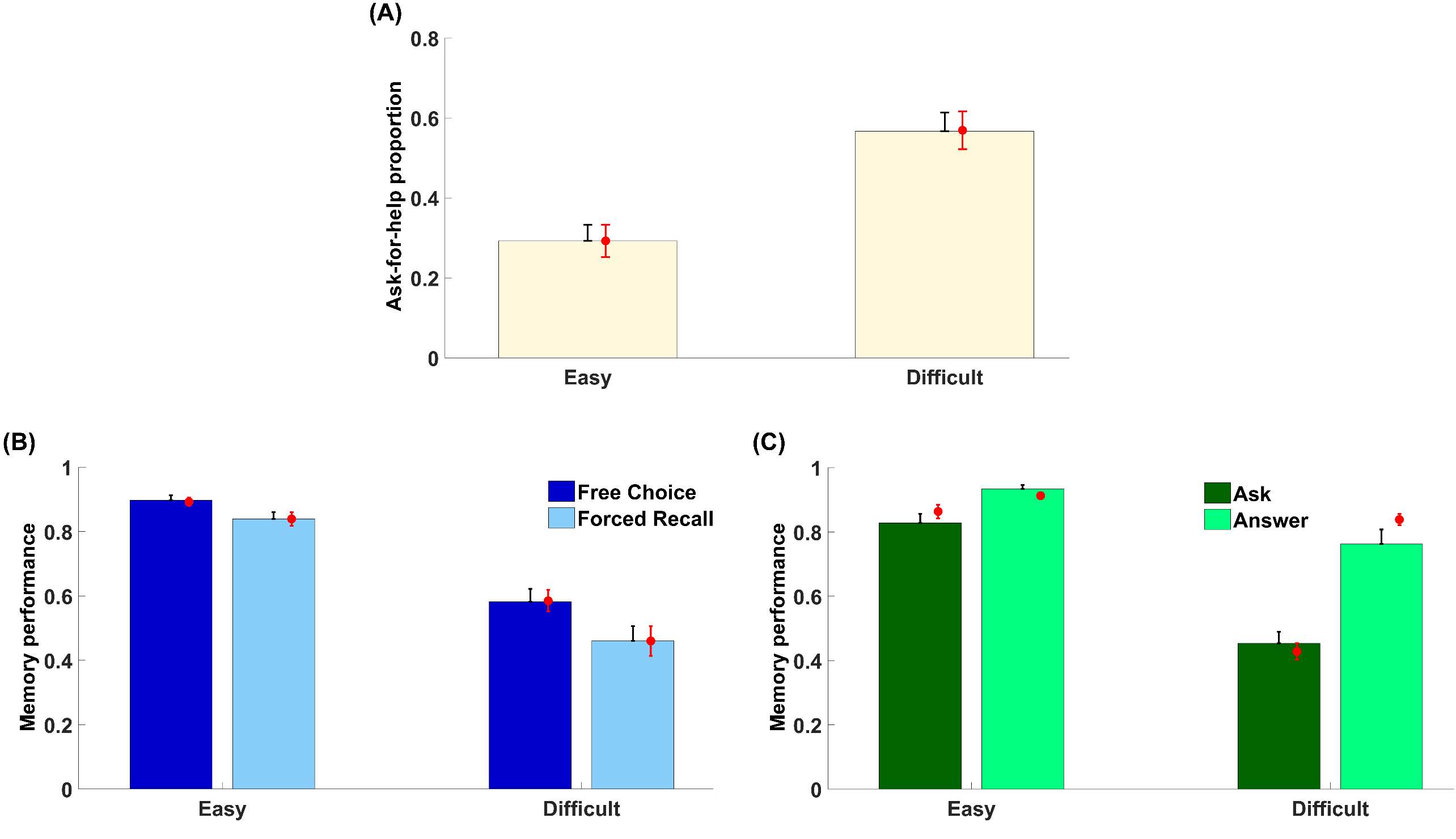
Figure S4.** Ask-for-help behaviour and memory performance in the memory test of Experiment 3. (A) Proportion of ask-for-help trials in the free-choice test was affected by item difficulty. (B) Memory performance was significantly higher in the free-choice than forced-recall test. (C) Memory performance in the free-choice test as a function of item difficulty and whether participants asked for help. The red points represent posterior predictives from the best-fitting computational model. Error bars represent standard errors.

**
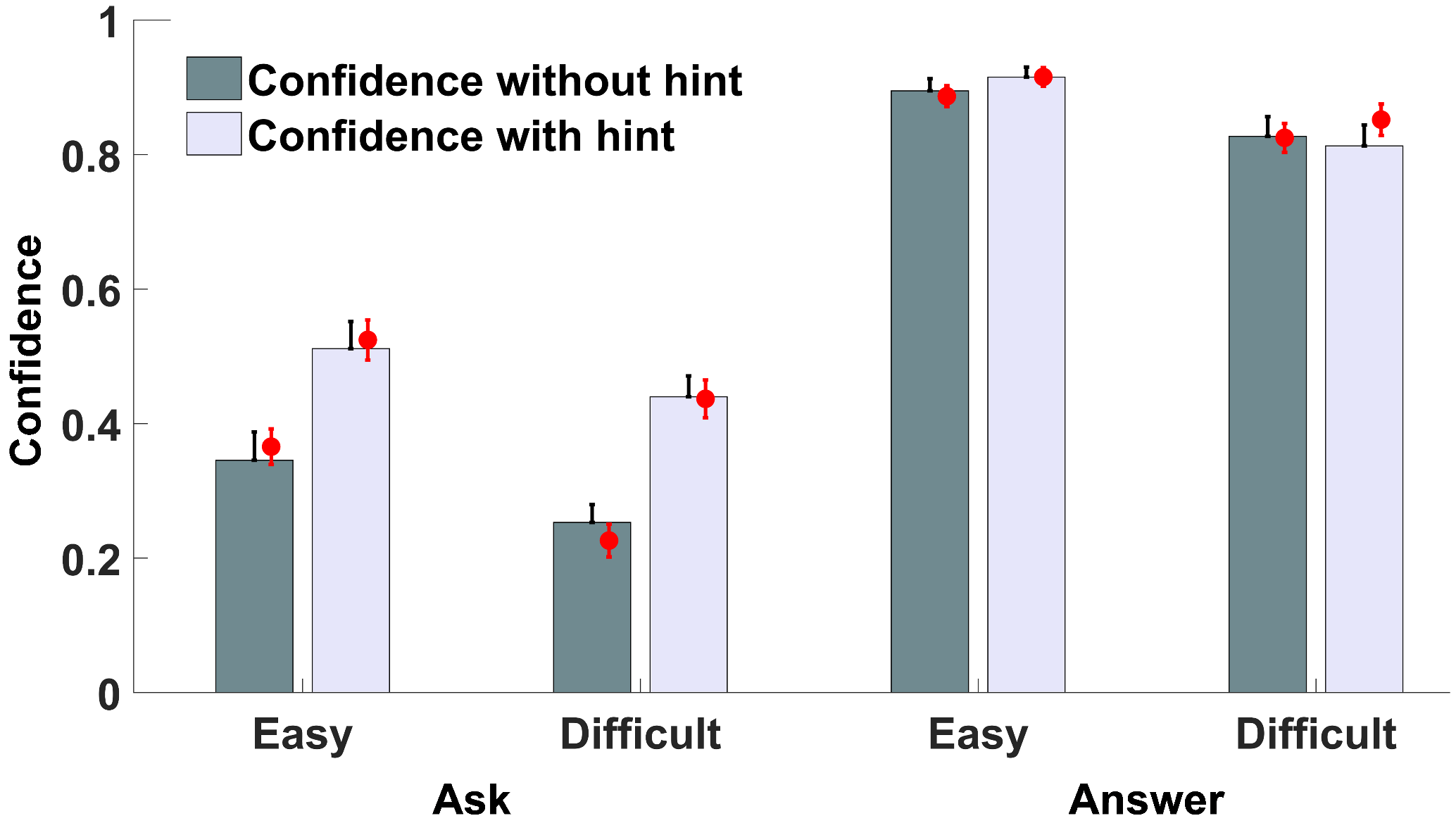
Figure S5**. Mean confidence ratings in the free-choice test of Experiment 3 as a function of answer/ask-for-help, whether the confidence was for trials with/without a hint, and item difficulty. Confidence was higher when participants chose to answer by themselves, and significantly modulated by the potential benefit of the hint only when participants chose to ask for help. The red points represent posterior predictives from the best-fitting computational model. Error bars represent standard errors.

**
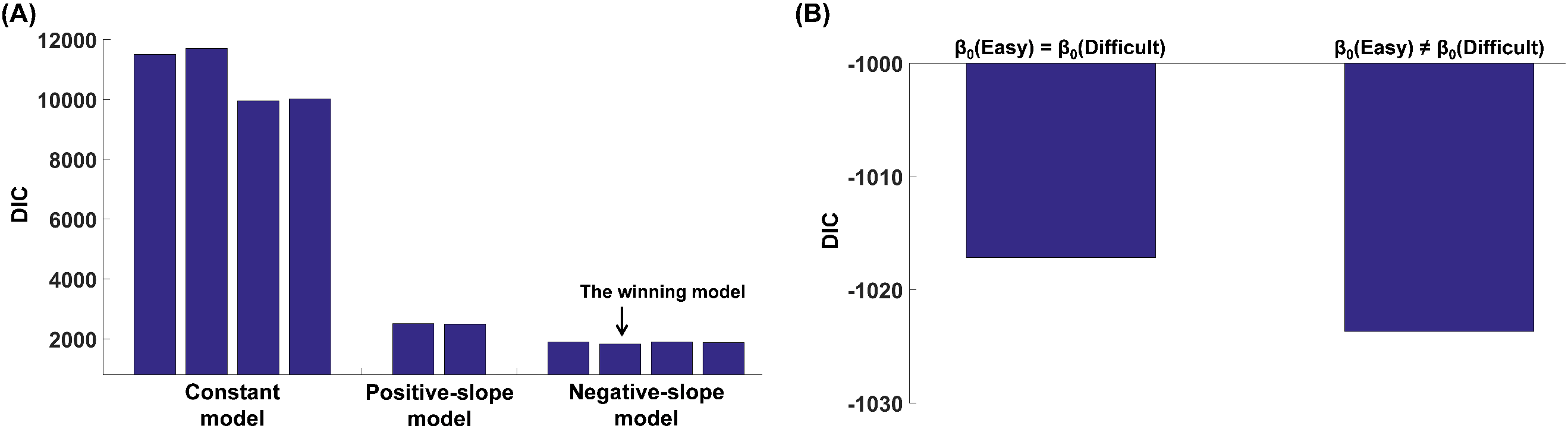
Figure S6**. The DIC scores of alternative models (lower is better) of the data from: (A) recall performance and decisions to ask for help in Experiment 3; (B) confidence ratings in Experiment 3.
